# Real-Time Tracking of Hot Carrier Injection at the Interface of FAPbBr_3_ Perovskite Using Femtosecond Mid-IR Spectroscopy

**DOI:** 10.1021/acscentsci.3c00562

**Published:** 2023-11-03

**Authors:** Issatay Nadinov, Khulud Almasabi, Luis Gutiérrez-Arzaluz, Simil Thomas, Bashir E. Hasanov, Osman M. Bakr, Husam N. Alshareef, Omar F. Mohammed

**Affiliations:** † Advanced Membranes and Porous Materials Center, Division of Physical Science and Engineering, King Abdullah University of Science and Technology, Thuwal 23955-6900, Kingdom of Saudi Arabia; ‡ Materials Science and Engineering, Physical Science and Engineering Division, King Abdullah University of Science and Technology (KAUST), Thuwal 23955-6900, Kingdom of Saudi Arabia; § Catalysis Center, Physical Science and Engineering Division, King Abdullah University of Science and Technology (KAUST), Thuwal 23955-6900, Kingdom of Saudi Arabia

## Abstract

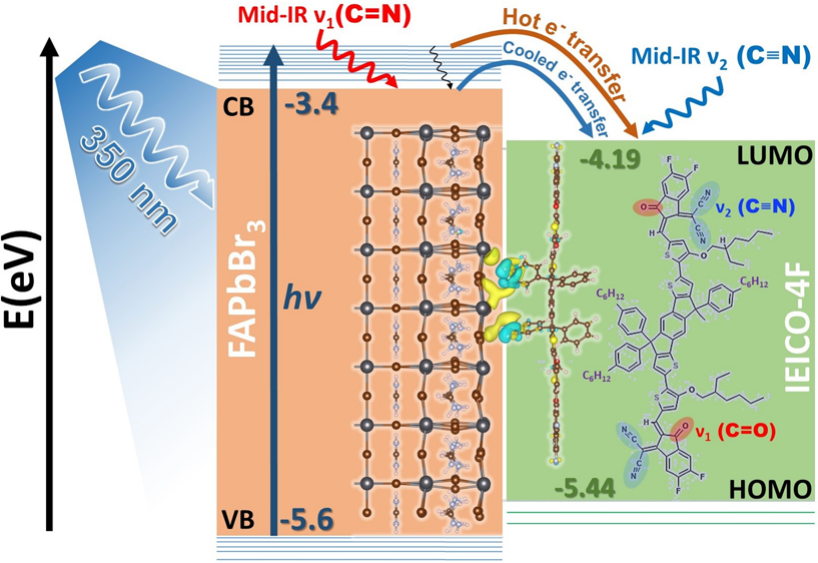

One of the most effective approaches to optimizing the performance of perovskite solar cells is to fully understand the ultrafast carrier dynamics at the interfaces between absorber and transporting layers at both the molecular and atomic levels. Here, the injection dynamics of hot and relaxed charge carriers at the interface between the hybrid perovskite, formamidinium lead bromide (FAPbBr_3_), and the organic electron acceptor, IEICO-4F, are investigated and deciphered by using femtosecond (fs) mid-infrared (IR), transient absorption (TA), and fluorescence spectroscopies. The visible femtosecond-TA measurements reveal the generation of hot carriers and their transition to free carriers in the pure FAPbBr_3_ film. Meanwhile, the efficient extraction of hot carriers in the mixed FAPbBr_3_/IEICO-4F film is clearly evidenced by the complete disappearance of their spectral signature. More specifically, the time-resolved results reveal that hot carriers are injected from FAPbBr_3_ to IEICO-4F within 150 fs, while the transfer time for the relaxed carriers is about 205 fs. The time-resolved mid-IR experiments also demonstrate the ultrafast formation of two peaks at 2115 and 2233 cm^–1^, which can be attributed to the C≡N symmetrical and asymmetrical vibrational modes of anionic IEICO-4F, thus providing crystal clear evidence for the electron transfer process between the donor and acceptor units. Moreover, photoluminescence (PL) lifetime measurements reveal an approximately 10-fold decrease in the donor lifetime in the presence of IEICO-4F, thereby confirming the efficient electron injection from the perovskite to the acceptor unit. In addition, the efficient electron injection at the FAPbBr_3_/IEICO-4F interface and its impact on the C≡N bond character are experimentally evidenced and align with density functional theory (DFT) calculations. This work offers new insights into the electron injection process at the FAPbBr_3_/IEICO-4F interface, which is crucial for developing efficient optoelectronic devices.

## Abstract 2

This study explores and deciphers ultrafast hot electron dynamics at the perovskite−electron-transporting layer interface using femtosecond visible and mid-IR spectroscopy.

## Introduction

Organic–inorganic hybrid perovskite materials have become increasingly popular in recent years due to their exceptional optical properties, including strong absorption coefficients of up to 10^5^ cm^–1^,^1−3^ high carrier mobilities of up to 1000 cm^2^ V^–1^ s^–1^,^4−7^ long carrier lifetimes of up to 4.5 μs,^6,8−10^ and long carrier diffusion lengths of up to 175 μm.^4,11^ These and other exceptional properties make these materials extremely competitive in various applications including lasers,^12^ solar cells,^13^ and light-emitting diodes (LEDs).^14−17^ Recent developments in the large-scale fabrication of organic–inorganic hybrid perovskite solar cells have simplified the manufacturing process and reduced the associated costs, thus providing a promising technology for the mass production of photovoltaic cells.^18,19^ Nevertheless, despite recent advances in perovskite solar cell (PSC) technology, the most efficient PSC (with an efficiency of over 25%) still faces significant challenges in practical applications. The stability and long-term reliability of PSCs remain major concerns that must be addressed before they can effectively compete with, and replace, existing photovoltaic technologies.^20−22^ To enhance the performance and the stability of the PSC, it is essential to select an appropriate electron donor–acceptor pair in order to facilitate efficient charge separation and collection.^23^ This typically involves using a hybrid perovskite as the donor and an organic material as the acceptor in the PSC device. While these approaches are promising, further fundamental research is required in order to improve the development of the technologies and materials. In particular, a deeper understanding of the physical processes (specifically the electron and hole injection processes) at the interfaces of organic–inorganic systems can help in designing superior materials for high-performance photovoltaic devices.

Ultrafast mid-infrared (IR) spectroscopy is a powerful technique that provides unique insights into the evolution of structural properties by accessing the vibrational bands of the excited molecules in real time. This characteristic makes it ideal for studying the electron transfer process at material interfaces at both the molecular and atomic levels. For instance, mid-IR spectroscopy has been used to study the carrier dynamics in metal complexes,^24^ organic-dye-sensitized solar cells,^25−29^ and semiconductors,^30−32^ thereby providing valuable dynamical information regarding the ultrafast injection and relaxation of the excited states of these materials. In other words, with its ability to probe the vibrational states of the reactants and products simultaneously, mid-IR spectroscopy is a useful tool that can provide an enhanced understanding of the intricate dynamics of charge transfer in various material systems. For instance, recent work has demonstrated the effectiveness of mid-IR spectroscopy in assigning the trapping process in perovskite materials.^33^ In addition, the vibrational charge dynamics and electron transfer in donor–acceptor systems containing organic molecules have also been studied using femtosecond (fs) techniques, as discussed by the John B. Asbury group.^34−40^ However, although visible and near-IR fs-transient absorption (TA) studies have been used more frequently to investigate and decipher charge transfer phenomena in perovskite-donor/organic-acceptor systems, they still do not fully capture the charge distribution in both materials upon excitation and its impact on molecular structure.^41,42^ By contrast, mid-IR spectroscopy is a powerful technique that can reveal the hidden processes of charge carrier transfer and provide a deeper understanding of these complex dynamics at the molecular level. Therefore, the use of mid-IR spectroscopy is particularly suitable for studying charge carrier transfer in these systems.

In the present study, the electron injection dynamics at the interface of the organic–inorganic hybrid perovskite, formamidinium lead bromide (FAPbBr_3_), and the organic electron acceptor, IEICO-4F (the chemical structure is shown in Figure S1a and b), are investigated by using fs-mid-IR spectroscopy to follow the ν(C≡N) and ν(C=O) vibrational modes of the IEICO-4F (acceptor) and the ν(C=N) vibrational mode of the FAPbBr_3_ (donor). It should be noted that the IEICO-4F molecule exhibits the potential for generating infrared-active vibrational bands, primarily due to its distinctive A–D–A (acceptor–donor–acceptor) molecular architecture.^43,44^ When the donor material is selectively excited at 350 nm, the generation and effective transfer of hot carriers to the organic acceptor material are observed within a femtosecond time scale. In addition to its properties as a perfect electron acceptor in recent PSC designs^45^ and its promising candidacy for use in ternary devices,^46^ the IEICO-4F material has well-isolated CN and CO vibrational modes. The fs-mid-IR transient absorption reveals an electron injection time of <150 fs at the FAPbBr_3_/IEICO-4F interface. Furthermore, the ultrafast carrier generation and transfer from the perovskite to the electron transporting layer are confirmed via an fs-TA study in the visible range after excitation at 350 nm (“hot” carriers) and 450 nm. In addition, the electron transfer process is supported by DFT calculations for the IEICO-4F in its ground, excited, and anionic states as well as for the FAPbBr_3_/IEICO-4F interface, thereby enabling the values of the vibrational modes of the donor–acceptor system to be extracted. The calculations reveal that electron injection weakens the C≡N bond character in the acceptor material, as evidenced by the extracted values of the vibrational spectra after excited-state electron transfer at the interface between the donor and acceptor moieties. Overall, the present work sheds light on the electron injection process at the FAPbBr_3_/IEICO-4F interface by using a combination of spectroscopic and theoretical techniques, thus providing important insights into the development of more efficient optoelectronic devices.

## Results and Discussion

### XRD, Steady-State Absorption, and Time-Resolved PL Experiments

**Figure 1:**
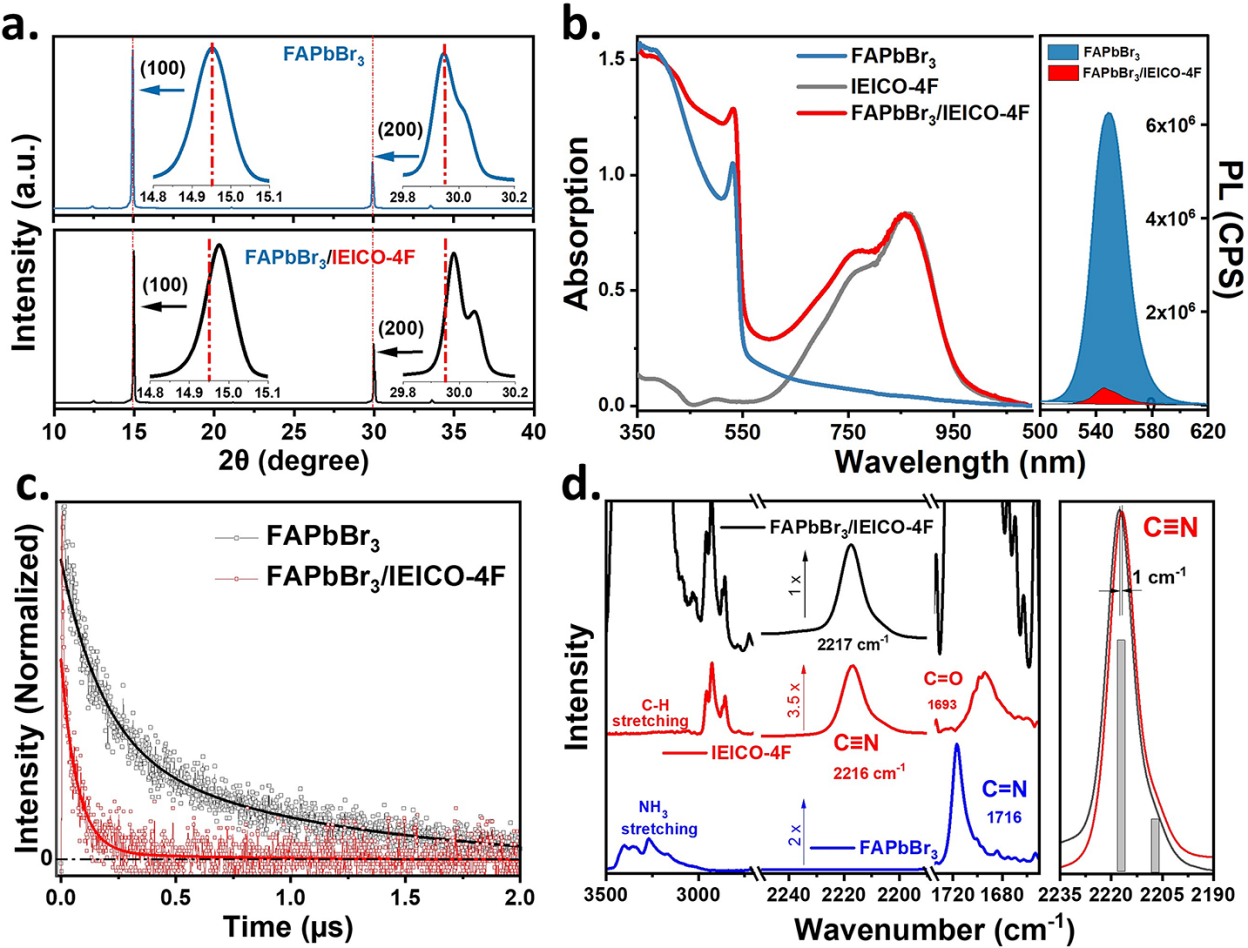
(a) XRD patterns of the FAPbBr_3_ (top) and FAPbBr_3_/IEICO-4F (bottom) films. (b) UV–NIR absorption and emission spectra of the FAPbBr_3_, IEICO-4F, and FAPbBr_3_/IEICO-4F films. (c) PL decay curve of the FAPbBr_3_ (black) and FAPbBr_3_/IEICO-4F (red) films, where the solid lines are the exponential fittings. (d) Steady-state FT-IR spectra of the FAPbBr_3_ (blue profile), IEICO-4F (red profile), and FAPbBr_3_/IEICO-4F (black profile) films, with the ν(C≡N) vibrational mode of IEICO-4F shown on the right.

The crystal structures of the FAPbBr_3_ and FAPbBr_3_/IEICO-4F films are revealed by the XRD results in Figure 1a. Here, FAPbBr_3_ and mixture FAPbBr_3_/IEICO-4F films exhibit a cubic structure belonging to the *Pm*3̅*m* space group, as indicated by the main XRD peaks at 14.95 and 29.95° due to the (100) and (200) diffraction planes, along with much weaker peaks at 21.2 and 33.6° due to the (110) and (210) diffraction planes of the FAPbBr_3_ structure.^47^ The dominance of the (100) and (200) crystal planes can be attributed to the 2D design of crystal symmetry. In addition, the FAPbBr_3_/IEICO-4F film exhibits a slight shift in the XRD pattern relative to that of the FAPbBr_3_ film, which can be attributed to shrinkage of the unit cell volume, and the resultant change in the lattice constant due to the incorporation of the charge acceptor. The extracted lattice parameters of the FAPbBr_3_ and FAPbBr_3_/IEICO-4F films are 5.952 and 5.946 Å, respectively. Interestingly, the above XRD analysis does not reveal any change in the FAPbBr_3_ crystal structure following exposure to IEICO-4F.

The optical properties of the FAPbBr_3_, IEICO-4F, and FAPbBr_3_/IEICO-4F films are revealed by the UV-NIR adsorption and emission spectra shown in Figure 1b. It should be noted that we can observe the characteristic absorption peak at 550 nm for FAPbBr_3_ and the mixture, and IEICO-4F exhibits peaks at 750 and 900 nm. It is worth mentioning that the absorption tail (550–800 nm) in FAPbBr_3_ could be attributed to the electron–phonon coupling of carriers below the band edge.^48,49^ Here, both films exhibit very narrow emission bands at 550 nm as well as strong quenching after excitation at 400 nm. These results are consistent with other optical studies on such donor–acceptor materials.^50,51^ Interestingly, the PL intensity of the FAPbBr_3_/IEICO-4F film is about 17 times lower than that of the FAPbBr_3_ alone (Figure 1b, right), thus indicating significant electron injection at the donor–acceptor interface.^52^

Further insight into the origin of the changes in PL intensity can be gained by comparing the PL lifetimes of the FAPbBr_3_ and FAPbBr_3_/IEICO-4F films in Figure 1c. Here, both films exhibit biexponential decay with two time constants, namely, τ_1_ = 137 ± 8 ns (60%) and τ_2_ = 980 ± 30 ns (40%) for the pure FAPbBr_3_ film and τ_1_ = 9 ± 2 ns (60%) and τ_2_ = 96 ± 10 ns (40%) for the FAPbBr_3_/IEICO-4F film. These time components reflect the charge carrier (electron/hole) recombination via the PL process on the surface and in the bulk film.^53^ The decrease in the PL lifetime constant of the donor (FAPbBr_3_) is consistent with efficient steady-state PL quenching, which can be attributed to charge transfer from the donor to the acceptor (IEICO-4F). It is important to note that charge injection occurs on a subpicosecond time scale, as clearly demonstrated by femtosecond mid-IR experiments (referenced below). However, due to the temporal resolution constraints of the time-correlated single photon counting (TCSPC) technique, only those contributions from the long-lived photoluminescence (PL) lifetime can be detected. These contributions might be attributed to long-distance charge transfer and recombination processes occurring on the nanosecond time scale.^54^

The presence of organic FA cations and IEICO-4F in the perovskite framework is confirmed by the FTIR results in Figure 1d. Here, the FAPbBr_3_ film (blue profile) exhibits a well-pronounced and intense peak at 1716 cm^–1^ that can be assigned to the ν(C=N) vibrational mode. Meanwhile, the IEICO-4F film (red profile) exhibits a broad peak at 1693 cm^–1^ due to the ν(C=O) stretching vibrational mode. Furthermore, a peak centered at 2216 cm^–1^ with a small shoulder at lower wavenumbers is observed in the range of 2150–2240 cm^–1^, corresponding to the symmetrical and asymmetrical ν(C≡N) vibrational modes, respectively. The veracity of these peaks and their corresponding assignments are corroborated by DFT calculations. In particular, the ν(C=O) stretching vibrational mode exhibits a shift of 5 cm^–1^ toward lower wavenumbers from the ground state to the excited state and a shift of 13 cm^–1^ from the ground state to the anionic state (refer to Figure S1c for visual representation). This indicates a reduction in the strength of the C=O bond character in the excited state with the weakening effect becoming even more pronounced in the anionic state. Notably, the FA^+^ cation of the perovskite does not exhibit any vibrational peaks in this range, which makes it possible to monitor the ν(C≡N) vibrational mode of the IEICO-4F after electron injection, for the first time, from the perovskite to the electron transporting layer. In the high-frequency region, the overlap of quadruple IR peaks at 3170, 3270, 3349, and 3401 cm^–1^ in the pure FAPbBr_3_ (blue profile) and mixed FAPbBr_3_/IEICO-4F film (black profile) is attributed to the N–H vibrational stretching modes of the FA^+^ cations, and is most likely due to the hydrogen bonding of the Br^–^ anion with the FA^+^ cation (N–H···Br). This suggests a strong interaction between the organic cation and the inorganic perovskite lattice.

In view of the above-mentioned FTIR results, two IR spectral ranges can be considered for monitoring the photoinduced dynamics via fs-mid-IR measurements. The first range (1650–1750 cm^–1^) allows the simultaneous tracking of the donor ν(C=N) and acceptor ν(C=O) vibrational dynamics of FAPbBr_3_/IEICO-4F following selective excitation of the donor at 450 nm (Figure S2). However, the strong overlap between these peaks hinders the clear observation of electron injection. The second range (2050–2300 cm^–1^) features the ν(C≡N) vibrational modes of IEICO-4F at 2216 cm^–1^, as shown on the right in Figure 1d. Monitoring the fs-IR measurement of this vibrational marker mode will enable the accurate assignment of electron injection in the FAPbBr_3_/IEICO-4F system, even after selective excitation of FAPbBr_3_ (electron donor) at 450 nm. Tracking the building up and the decay of this vibrational mode is expected to reveal the speed of injection and charge recombination in the system, which will be reflected in changes in the vibrational peak positions in response to changes in the local environment.

### fs-TA Spectroscopy of the FAPbBr_3_ and FAPbBr_3_/IEICO-4F

**Figure 2:**
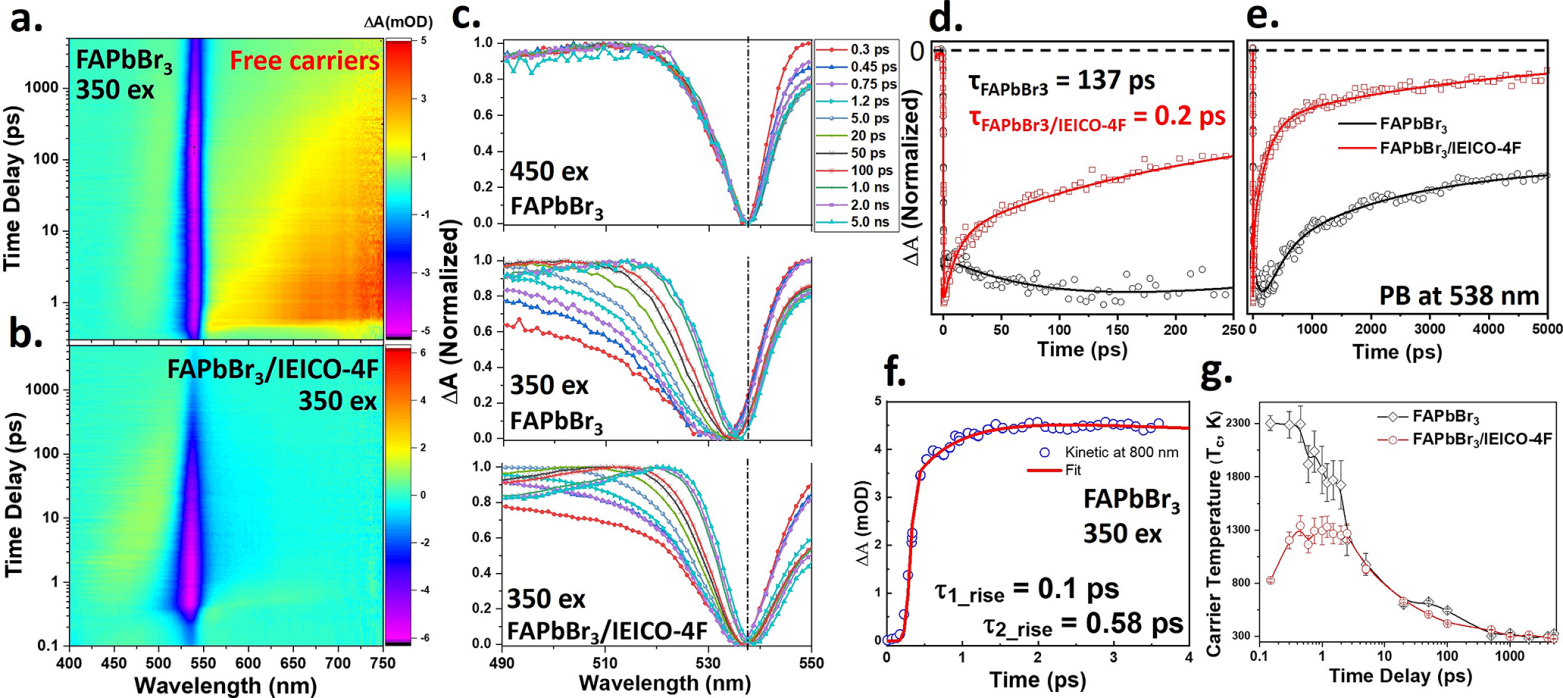
(a and b) Map plots of the fs-TA spectroscopic measurements for the (a) FAPbBr_3_ and (b) FAPbBr_3_/IEICO-4F films after excitation at 350 nm. (c) The normalized fs-TA spectra of the FAPbBr_3_ film after excitation at 450 nm (top) and 350 nm (middle) and of the FAPbBr_3_/IEICO-4F film after excitation at 350 mn (bottom). (d) Short-term and (e) long-term kinetic traces of the FAPbBr_3_ film (black) and the FAPbBr_3_/IEICO-4F film (red) monitored at 538 nm. (f) Kinetic trace of the free carriers in the FAPbBr_3_ film monitored at 800 nm. (g) Cooling dynamics of the hot carriers in the FAPbBr_3_ and FAPbBr_3_/IEICO-4F films as a function of time delay after excitation at 350 nm (extracted from part c).

The photoinduced charge injection process at the interface between the donor and acceptor system is elucidated by the map plots of the time-resolved fs-TA measurements within the UV–vis region following selective excitation of the perovskite material at 350 nm, as shown in Figure 2a and 2b. The TA spectra exhibit the following two key features: (i) a negative change (Δ*A* < 0) known as photobleaching (PB) and (ii) a positive change (Δ*A* > 0) known as photoinduced absorption (PIA). Both the FAPbBr_3_ film (Figure 2a) and the FAPbBr_3_/IEICO-4F film (Figure 2b) exhibit a strong PB signal (blue) at 538 nm, corresponding to the FAPbBr_3_ band gap. Notably, the broad, positive PIA signal (red) observed in the region of 550–750 nm for the pure FAPbBr_3_ film is absent in the case of the mixed film. This feature represents excited-state absorption and is attributed to the free carriers forming mainly from hot carriers upon excess energy excitation. The normalized fs-TA spectra of the FAPbBr_3_ film over a time scale of 0.3 ps to 5 ns after 450 and 350 nm excitations, along with that of the FAPbBr_3_/IEICO-4F film after 350 nm excitation, are presented in Figure 2c. Here, while the 450 nm excitation spectra remain unchanged over the entire time scale, the 350 nm excitations for both films exhibit a broadening and a shift in the PB signal toward the low-energy region (540–550 nm). These changes are attributed to the quasi-equilibrium distribution of hot carriers in the perovskite crystal lattice and the band gap renormalization process, respectively.^55,56^ In addition, a slight difference is observed between the PB peak shifts of the FAPbBr_3_ and FAPbBr_3_/IEICO-4F films, which can be explained by electronic coupling between the donor and acceptor at the interfaces, along with a small change in the lattice size of the perovskite material due to the introduction of IEICO-4F, as observed in the XRD pattern (Figure 1a). After ∼5 ps, a gradual narrowing of the bleach signal begins to appear in Figure 2c (for spectra under 350 nm excitation), which can be attributed to the hot carrier thermalization process.^57^ By contrast, few or no free carriers are formed in the FAPbBr_3_ film under 450 nm excitation, as shown in Figure S3, which supports the process of the formation of free carriers from hot carriers in response to high-energy excitation.

The kinetic fits at 538 nm in Figure 2d and 2e indicate exponential increases in the time constants of τ_1_ = 205 fs and 137 ps for the FAPbBr_3_/IEICO-4F and FAPbBr_3_ films, respectively. This can be attributed to the process of intraband relaxation (hot carrier cooling) and the intrinsic hot phonon bottleneck effect.^58^ The fast signal decay of the mixed film may be the first evidence of the efficient transfer of the hot carriers after their generation. Furthermore, the lack of formation of free carriers from hot carriers in the low-energy band for the mixture FAPbBr_3_/IEICO-4F (Figure 2b) confirms that hot carrier extraction occurs within ∼150 fs. It should be noted that the generation time of the free carriers from hot carriers in the FAPbBr_3_ film at 350 nm excitation can be extracted from the exponential fit of the rising kinetic trace at 800 nm in Figure 2f, which is found to be 0.58 ps.

The rates of hot carrier extraction in the FAPbBr_3_ and FAPbBr_3_/IEICO-4F films can be compared in terms of the calculated temperature dynamics of the carriers by fitting the normalized TA spectra in the high-energy tail range from 460 (2.7) to 535 nm (2.32 eV). It is known that hot carriers have excess energy, more than *k*_*B*_*T*, above the conduction band. Therefore, the carrier temperature (*T*_*C*_) follows the Maxwell–Boltzmann distribution function *ΔA*[*E*] ∝ exp^–(*E*–*E*_*F*_)/*k*_*B*_*T*_*C*_)^, where *ΔA* is the change in the TA absorption, *E*_*F*_ is the Fermi level, and *k*_*B*_ is Boltzmann’s constant.^41^ The single-exponential fittings of the high-energy tails in the normalized fs-TA spectra of the FAPbBr_3_ and FAPbBr_3_/IEICO-4F films after excitation at 350 nm are presented in Figure S4. The extracted *T*_*C*_ values as a function of time delay for the FAPbBr_3_ and FAPbBr_3_/IEICO-4F films are presented in Figure 2g. Here, while similar slow decays in the range of 2 to 2000 ps are observed for both films, the dynamics clearly differ in the very short term (0.15 to 2 ps). Specifically, the *T*_*C*_ for the pure FAPbBr_3_ film begins to decrease within temporal resolution immediately after excitation (<120 fs) with the initial temperature value of 2300 K, while the *T*_*C*_ of the FAPbBr_3_/IEICO-4F film increases during the time period of 150 to 300 fs to reach a saturation value of 1300 K before slowly decaying at the same rate as for the pure FAPbBr_3_. The early *T*_*C*_ rise time of the mixed film is attributed to the delayed equilibrium of the hot carriers.^42^ These results further demonstrate the femtosecond nature of hot carrier transfer in the donor–acceptor system of the FAPbBr_3_/IEICO-4F film after excitation at 350 nm.

It is reasonable to assume that the excess energy of the hot carriers is efficiently transferred from FAPbBr_3_ to IEICO-4F before it begins to thermalize to the crystal lattice of the perovskite. The second component of fitting (Figure 2e) reveals a time constant of >5 ns, which could be attributed to charge recombination. Moreover, the FAPbBr_3_/IEICO-4F film exhibits a faster decay than does the pure FAPbBr_3_ film. This suggests that charge separation and recombination mechanisms occur more rapidly in the donor–acceptor system than in pure perovskite. A dynamic study of the free and hot carriers injected from the perovskite into the electron-accepting material and of the structural changes induced during the transfer process is presented in the following section by using fs time-resolved mid-IR spectroscopy.

### Mid-IR fs Spectroscopy for FAPbBr_3_ and FAPbBr_3_/IEICO-4F

**Figure 3:**
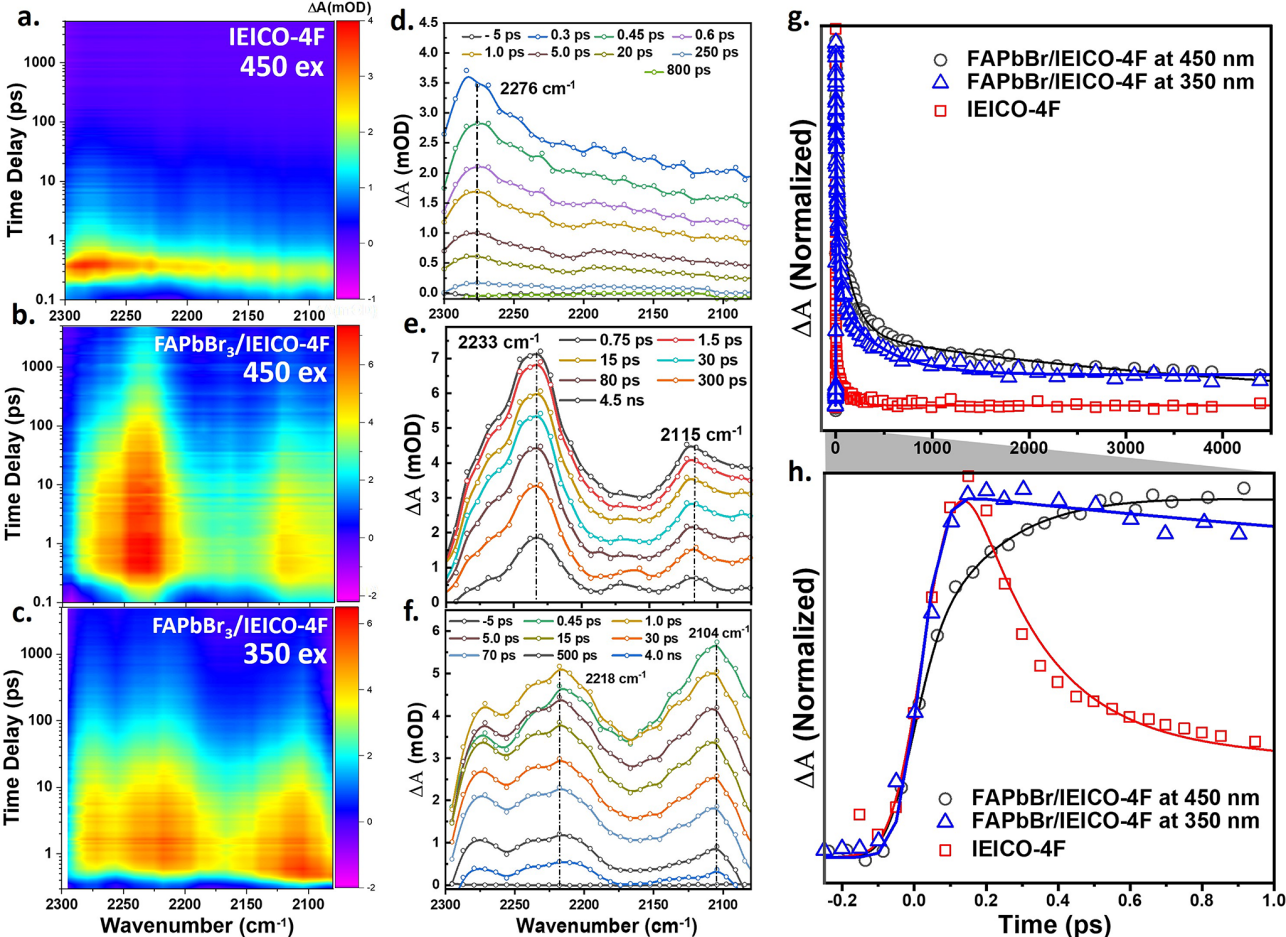
(a–c) Map plots of the mid-IR fs spectroscopic measurements for (a) the IEICO-4F ν(C≡N) film after excitation at 450 nm and (b and c) the FAPbBr_3_/IEICO-4F film after excitation at (b) 450 nm and (c) 350 nm. (d–f) Corresponding transient mid-IR spectra. (g and h) Kinetic traces of the transient mid-IR ν(C≡N) stretching vibration at 2276 cm^–1^ for the IEICO-4F excited at 450 nm (red) and the FAPbBr_3_/IEICO-4F excited at 450 nm (black) and 350 nm (blue) in the (g) long and (h) short time windows.

The transfer dynamics of the photogenerated carriers from the perovskite to the organic electron acceptor molecules are revealed by the map plots of the mid-IR fs spectroscopy measurements for the IEICO-4F film excited at 450 nm and those of the FAPbBr_3_/IEICO-4F film excited at 450 and 350 nm in Figure 3a–c, respectively. Here, the mid-IR spectrum of the IEICO-4F film (Figure 3a) exhibits a broad band at 2276 cm^–1^ due to the asymmetric ν(C≡N) stretch in the excited state of the neutral. Due to the weak absorption of the IEICO-4F film at a 450 nm excitation wavelength, we measured a transient mid-IR spectrum in the same region as shown in Figure 3a but under 730 nm excitation (Figure S5) to confirm the similarity of the spectra and exclude the multiphoton effects upon 450 nm excitation. In addition, we present the CN band centered in the 2180–2350 cm^–1^ range (Figure S6). As indicated in Figure 3d, a weak ground-state bleach signal appears on top of the broad positive peak at 2215 cm^–1^. The low intensity of the ground-state bleaching signal is caused by the strong overlap with the more intense excited-state absorption at 2276 cm^–1^. By contrast, the spectrum of the FAPbBr_3_/IEICO-4F film in Figure 3e exhibits two strong, relatively narrow positive peaks at 2115 and 2233 cm^–1^ after 450 nm excitation, attributed to the ν(C≡N) symmetrical and asymmetrical stretches of the anionic form of the acceptor, respectively. The red shift of these peaks compared to the neutral IEICO-4F provides evidence for the electron transfer from the perovskite to the IEICO-4F acceptor system.

The effect of the hot carriers on the ν(C≡N) vibrational marker mode in the mid-IR spectrum of the FAPbBr_3_/IEICO-4F film under 350 nm excitation is shown in Figure 3c and f. As can be seen, the two positive peaks due to the ν(C≡N) symmetrical and asymmetrical stretches are again observed, but they are broadened and more red-shifted to 2104 and 2218 cm^–1^, respectively, relative to those observed under the 450 nm excitation. The broadening can be attributed to the transfer of free carriers into the IECO-4F molecule, while the shift to the low wavenumber can be explained by the weakening of the ν(C≡N) bond due to the heat produced by the generation of hot carriers at the interface. Furthermore, the excess energy of the hot electrons speeds up the electron injection, which directly affects the build-up rate of the ν(C≡N) vibrational signal in the excited anionic state (Figure 3g and h). Additionally, the observed shift in the ν(C≡N) vibrational peaks is in line with calculated DFT results (next section).

Furthermore, a comparison of the kinetic trace at 2277 cm^–1^ for the IEICO-4F in the neutral state (red profile, Figure 3h) with that at 2115 cm^–1^ for the FAPbBr_3_/IEICO-4F under 450 nm excitation (black profile, Figure 3h, where the IEICO-4F takes the anionic form) and 350 nm excitation (blue profile, Figure 3h, where hot carriers are generated and transferred) reveals the different rates of electron transfer. More specifically, the selective excitation of the donor at 450 nm directly affects the buildup of the ν(C≡N) vibrational peaks of the anionic IEICO-4F, with a rising time of 1 ps, and can be attributed to the electron-transfer time constant. Notably, the very fast formation of the anionic species (<150 fs) upon excess energy excitation (350 nm) speeds up the electron transfer from the perovskite to the acceptor molecules.

### DFT Calculations

**Figure 4:**
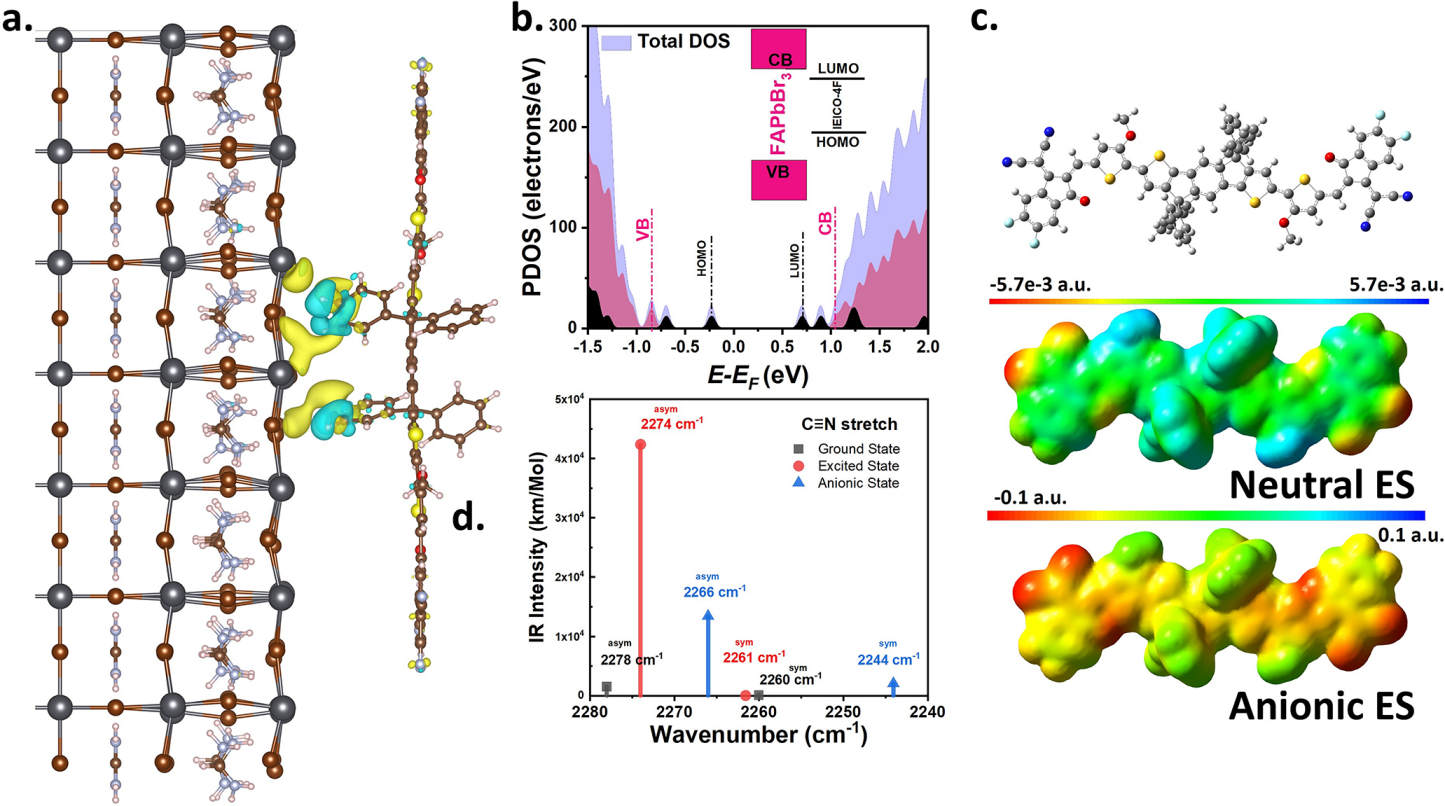
(a) Charge density difference plot when IEICO-4F is adsorbed on the FAPbBr_3_ [100] surface. (b) Projected spin-polarized electronic density of states (PDOS) of the IEICO-4F on the FAPbBr_3_ surface. (c) Molecular structure (top) and molecular electrostatic potential (MESP) maps for the excited states of the neutral (middle) and anionic (bottom) forms of the IEICO-4F molecule. (d) Calculated IR vibrational spectra for the excited and anionic states of IEICO-4F, where “asym” and “sym” correspond to asymmetrical and symmetrical vibrational modes, respectively.

The interface between the IEICO-4F and the FAPbBr_3_ is shown in Figure 4a and can be analyzed according to the difference in charge density between the hybrid and individual components as
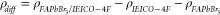
where *ρ*_*FAPbBr*___3_/*IEICO*-4*F*_, *ρ*_*IEICO*__–4*F*_, and *ρ*_*FAPbBr*_3__ are the total and individual charge-density components of the IEICO-4F and FAPbBr_3_, respectively. An analysis of the optimized structure shows that the structural relaxations mostly affect the interfacial region and deformations do not extend far from the interface (Figure 4a). Here, the side chain of the IEICO-4F (alkyl substituted phenyl ring) is closest to the FAPbBr_3_ surface, and the shortest distance is 2.85 Å (between the H atom of IEICO-4F and the Br atom of FAPbBr_3_), which generates the binding energy of 0.86 eV between two systems. Furthermore, the extent of charge transfer across the interface is quantified via Bader charge analysis,^59^ which indicates a transfer of 0.29 electrons from the FAPbBr_3_ to the IEICO-4F. This is also evident in the charge-density difference plots in Figure 4a. Furthermore, the projected density of states (PDOS) of IEICO-4F on the FAPbBr_3_ surface in Figure 4b reveals that the HOMO and LUMO levels (black dotted lines) of the organic molecule fall within the band gap of the perovskite material (red dotted lines). However, the HOMO level of IEICO-4F is located far from the valence band maximum of FAPbBr_3_, thereby posing a challenge for hole transfer at this interface. By contrast, the LUMO + 1 level of the IEICO-4F is in close proximity to the conduction band of the perovskite, thereby allowing for more efficient electron injection from the perovskite to the IEICO-4F.

The molecular electrostatic potential (MESP) is plotted for both the neutral and anionic forms of the IEICO-4F in Figure 4c, where color bars are given in atomic units (a.u.). As expected, the MESP isosurface of the anion is seen to be much more negative (red/yellow) than the neutral system (mostly green/cyan). Furthermore, even the ν(C≡N) region of the anionic state exhibits a higher negative charge than that in the neutral state, which affects the bond character. Indeed, the weakening of the C≡N bond character of the acceptor in the anionic state is revealed by the ν(C≡N) vibrational peaks extracted from the IR spectra (Figure 4d). Here, the symmetrical and asymmetrical ν(C≡N) vibrational peaks of the anionic state (red profile) are seen to be downshifted by 8 and 17 cm^–1^, respectively, compared to the neutral state (black profile). These observations, including the peak shift, are consistent with the above-mentioned mid-IR spectral measurements. Note that our B3LYP-calculated vibrational frequencies deviate from experimental values due to the lack of anharmonicity in the model as reported in the literature.^60^

Considering the DFT results for the interface between FAPbBr_3_ and IEICO-4F, it can be concluded that electron injection originates between the alkyl-substituted phenyl ring of the IEICO-4F and FAPbBr_3_ lattice. Additionally, the DFT calculations for the acceptor in the neutral and anionic states indicate a change in the electronic density distribution, leading to a weakening of the C≡N bond character due to the injection of electrons and the formation of the anionic species.

## Conclusions

The efficient extraction of hot carriers at the interface between an organic–inorganic hybrid perovskite FAPbBr_3_ and an organic electron acceptor IEICO-4F was demonstrated herein via a combination of visible and mid-infrared (IR) femtosecond (fs) techniques along with density functional theory (DFT) calculations. These techniques, especially fs mid-IR, were selected for their ability to provide detailed information about the interface between the materials that cannot be directly obtained through traditional optical techniques. The time-resolved results indicated that the hot carriers are generated in the pure FAPbBr_3_ film and their transition to free carriers occurs within 150 fs, whereas the hot carriers in the hybrid FAPbBr_3_/IEICO-4F system are transferred to the electron-accepting unit to generate the anionic form of IEICO-4F within a femtosecond time scale. In addition, the fs-mid-IR technique made it possible to follow the distinct spectral and dynamical changes in the ν(C≡N) vibrational marker mode in the neutral and anionic species of the IEICO-4F molecule. Interestingly, both the fs-TA and fs-mid IR results clearly demonstrated that the excess energy of the hot carriers shortens their injection time compared to that of the relaxed carriers. Furthermore, the DFT calculations highlighted the changes in the charge delocalization and the weakening of the ν(C≡N) vibrational band due to the formation of the radical anion upon electron transfer to the acceptor. Thus, the present findings provide an enhanced understanding of the structural and electronic properties of these materials, along with useful insights for the development of new perovskite-based materials that are more efficient in light energy conversion.

## Experimental Methods

### FAPbBr_3_ Film Fabrication

#### Materials

Lead bromide (PbBr_2_, 99.99%) was purchased from Tokyo Chemical Industry Co. (TCI). Formamidinium bromide (FABr, >99%) was purchased from Greatcell Solar (Australia). The nonfullerene acceptor (NFA) material IEICO-4F was purchased from 1-Material. Dimethylformamide (DMF, >99.8%), dimethyl sulfoxide (DMSO, >99.8), and chlorobenzene (CB, >99.8%) were purchased from Acros Organics (Belgium). Anhydrous toluene (99.8%) was purchased from Sigma-Aldrich (USA). The 25-mm-diameter calcium fluoride (CaF_2_) substrates were obtained from China and cleaned via consecutive sonication in detergent, deionized water, acetone, and isopropyl alcohol for 10 min each. This was followed by ultraviolet ozone (UVO) surface treatment for 15 min prior to spin coating.

#### Preparation of the Perovskite Precursor Solution

A 1 M solution of FAPbBr_3_ in 9:1 DMF:DMSO was prepared by dissolving equimolar amounts of FABr and PbBr_2_ in the mixed solvent and stirring overnight at room temperature. A 100 μL aliquot of the perovskite precursor was then filtered and spin-coated onto the CaF_2_ substrate at 1000 rpm for 30 s while dropping 150 μL of toluene. The substrates were then annealed at 100 °C for 10 min.

#### Preparation of the IEICO-4F Films

A 20 mg/mL solution of IEICO-4F in CB was prepared and stirred overnight at 60 °C and then filtered. A 100 μL aliquot of the solution was then spin-coated onto the CaF_2_ substrate at 1000 rpm for 30 s, followed by annealing at 110 °C for 10 min.

#### Preparation of the Mixed FAPbPBr_3_/IEICO-4F Layers

The same procedure of spin-coating the perovskite precursor and annealing was followed by the spin-coating of a layer of IEICO-4F and annealing to remove the excess solvent. These two consecutive procedures were then repeated to obtain four layers in total.

The crystal structures of the FAPbBr_3_ and FAPbBr_3_/IEICO-4F films were first examined with a Bruker D8 Advance diffractometer using Cu Kα radiation (λ = 1.5418 Å) in the 2θ range of 5–50° at a 2θ step size of 0.02° and a scanning rate of 2 deg/min. The film thickness was determined with the Tencor stylus profiler surface measurement system (Tencor *P*6̅, by applying a 0.5 mN force on the probing tip) and cross-sectional SEM images (see Figure S7). The thicknesses of the samples are found to be 673 nm for pure FAPbBr_3_ and 902 nm for mixture FAPbBr_3_/IEICO-4F, which are in good agreement with the thickness obtained from profiler measurements (610 and 1070 nm, respectively).

### Steady-State Absorption and Photoluminescence (PL) Measurements

The steady-state absorption and photoluminescence (PL) measurements were, respectively, acquired by using a Cary-5000 UV–vis–NIR spectrophotometer (Agilent) and a Fluoromax-4 spectrofluorometer (Horiba). The static infrared measurements were performed on an Agilent Cary 600 series Fourier transform infrared (FTIR) spectrometer with a spectral resolution of 0.1 cm^–1^. Steady-state measurements were obtained for the FAPbBr_3_, IEICO-4F, and mixed FAPbBr_3_/IEICO-4F films, with the CaF_2_ window in order to gain a comprehensive understanding of the optical properties of the composite material and its response to different excitation conditions.

### Time-Resolved Mid-Infrared Spectroscopy (fs-IR)

The time-resolved mid-IR experiments were performed using a Helios-IR spectrometer with broadband capability (Ultrafast Systems). The output pulses from a 150 fs Ti:sapphire regenerative amplifier operating at 1 kHz and 800 nm (Astrella laser system, Coherent Inc.) were split in order to drive two near-IR spectrally tunable optical parametric amplifiers (TOPAS Prime; Light Conversion/Spectra-Physics). The first portion of light was used to generate the visible pump pulses at 400 and 750 nm, and the second portion was used for tunable mid-IR probe pulse generation via the difference frequency mixer. To study the ν(C=N) vibrational mode of the FAPbBr_3_ and the ν(C=O) and ν(C≡N) vibrational modes of the IEICO-4F, the selected spectral window for these measurements was 1650–3500 cm^–1^. For the fs-mid-IR measurements, both the pump and probe pulses were directed and overlapped at the sample and the transmitted mid-IR probe light was detected using a charge-coupled device (CCD) that was cooled by liquid nitrogen (N_2_). Transient IR measurements were conducted for the spin-coated FAPbBr_3_, the IEICO-4F, and the mixed FAPbBr_3_/IEICO-4F films on the CaF_2_ substrates. The fs-mid-IR measurements were performed under N_2_ gas conditions to minimize any potential interference from the air. Finally, to ensure accurate and reliable measurements with minimum photodegradation, a translating sample holder was used to rotate the samples during the measurements so that a fresh area of the film could be excited at each laser shot. The FAPbBr_3_ and FAPbBr_3_/IEICO-4F films were excited at 450 and 350 nm with a fixed optical pump fluence of 4 μJ/cm^2^ and an excited beam spot size (diameter) of ∼0.03 cm and probed with a broad mid-IR pulse in the range of 2050 to 2300 cm^–1^. The excitation wavelength of 450 nm was chosen for the mixed coating in order to maintain the absorption contribution of the IEICO-4F at a negligible level (under 1%); this is termed selective excitation of the FAPbBr_3_ in the composite.

### Time-Resolved Spectroscopy in the Visible Range (fs-TA)

The fs-TA measurements were obtained on a Helios spectrometer. For this purpose, the film samples were excited with pump pulses at 350 and 450 nm with a fixed optical pump fluence of 4 μJ/cm^2^ and an excited beam spot size (diameter) of ∼0.02 cm, which were generated after passing through a fraction of an 800 nm beam (Astrella, Coherent, ∼150 fs pulse, 1 kHz, 7 mJ/pulse) into a spectrally tunable optical parametric amplifier (TOPAS, Newport Spectra-Physics). The probe pulses (UV visible and NIR wavelength continuum, white light) were generated by passing another fraction of the 800 nm pulse through a 2-mm-thick CaF_2_ crystal. Before the white light was generated, the 800 nm amplified pulses were passed through a motorized delay stage. Depending on the movement of the delay stage, the transient species were detected following excitation at different time delays. The white light was split into two beams (signal and reference) and focused on two fiber optic devices to improve the signal-to-noise ratio. The excitation pump pulses were spatially overlapped with the probe pulses on the samples after passing through a synchronized mechanical chopper (500 Hz) which blocked alternate pump pulses. The absorption change (Δ*A*) was measured based on the time delay and wavelength (λ). The IRF for the TA was measured to be 168 fs.

### Time-Resolved Photoluminescence (PL)

For the time-resolved PL experiments, the FAPbBr_3_ and mixed FAPbBr_3_/IEICO-4F films were excited at 350 nm by a pulsed diode laser (90 ps, Horiba, Delta Diode) focused through the 20×, 0.38 NA objective of a modified microscope (Olympus IX71). The interpulse duration was set to be longer than the PL decay time to ensure complete relaxation (10 MHz), and the intensity of the pulses was adjusted using a set of neutral density filters (Thorlabs) to ensure that less than 1% of the excitation events resulted in the detection of a single photon. A long-pass 490 nm filter (Newport) was used to reject the scattered laser light and to select the proper emission wavelength to be monitored. The filtered PL signal was directed and focused on an avalanche photodiode (PDM series, MicroPhoton Devices), and the time-correlated single-photon counting (TCSPC) data were collected by using a HydraHarp 400 controller (PicoQuant). The overall time resolution of the system was better than 200 ps. The histograms obtained were fitted with SymphoTime64 software (PicoQuant) using the Levenberg–Marquardt iteration algorithm.

### Computational Methods

The geometric optimization of the adsorbate on the FAPbBr_3_ surface was performed using the CP2K software (version 9.1)^61^ with Goedecker–Teter–Hutter (GTH) pseudopotentials in combination with DZVP-MOLOPT-SR-GTH basis sets. A plane-wave cutoff of 300 Ry was used across four grids, with a relative cutoff of 40 Ry. The CP2K calculations were spin-unpolarized and performed at the Γ-point only for a (7 × 3) unit cell of FAPbBr_3_. In the geometric optimization, the atoms on the top three layers of the supercell were allowed to relax, and the remaining atoms in the other layers were fixed at their bulk-optimized geometries. The geometries were optimized via the Broyden–Fletcher–Goldfarb–Shanno (BFGS) method until the maximum force was less than 4 × 10^–4^ Ha Bohr^–1^. The Perdew–Burke–Ernzerhof (PBE) exchange-correlation functional was combined with Grimme’s D3 dispersion method and Becke–Johnson (BJ) damping to account for the van der Waals interactions. Further single-point calculations, including the Bader charge and density of states (DOS), were performed using the Vienna ab initio simulation package (VASP).^62^ The DFT calculations for the IEICO-4F molecule were performed using the Gaussian 16, revision C.02 suite of programs^63^ and the 6-31G* basis. The B3LYP functionals were considered, and the influence of the dielectric constant was modeled (taking into account a dielectric constant, ε, of 4.71) by using the integral equation formalism of the polarizable continuum model (IEF-PCM), a solvation model within the self-consistent reaction field (SCRF) framework. Finally, unscaled harmonic vibrational frequencies are presented. These frequencies were used to confirm that the geometry corresponds to a minimum-energy state.

## Supporting Information Available

Chemical structures; map plots; spectra; and single-exponential fitting (PDF)
Transparent Peer Review report available (PDF)
