# Supplementary material for: Real-Time Tracking of Hot Carrier Injection at the Interface of FAPbBr3 Perovskite Using Femtosecond Mid-IR Spectroscopy
Source: ACS Cent Sci. 2023 Nov 3;10(1):43–53. doi: 10.1021/acscentsci.3c00562 (PMC10823510; doi:10.1021/acscentsci.3c00562)
Supplement: Supplementary file 1 — oc3c00562_si_001.pdf [file oc3c00562_si_001.pdf]

Supporting Information

Real-Time Tracking of Hot Carrier Injection at the Interface of $FAPbBr_3$ Perovskite Using Femtosecond Mid-IR Spectroscopy

Issatay Nadinov,[1,2] Khulud Almasabi,[3] Luis Gutiérrez-Arzaluz,[1,3] Simil Thomas,[1] Bashir E. Hasanov,[3] Osman M. Bakr,[3] Husam N. Alshareef,[2] Omar F. Mohammed* [1,3]

*email: Omar.Abdelsaboor@kaust.edu.sa

[1]Advanced Membranes and Porous Materials Center, Division of Physical Science and Engineering, King Abdullah University of Science and Technology, Thuwal 23955-6900, Kingdom of Saudi Arabia

[2]Materials Science and Engineering, Physical Science and Engineering Division, King Abdullah University of Science and Technology (KAUST), Thuwal 23955-6900, Saudi Arabia

[3]Catalysis Center, Physical Science and Engineering Division, King Abdullah University of Science and Technology (KAUST), Thuwal 23955-6900, Kingdom of Saudi Arabia

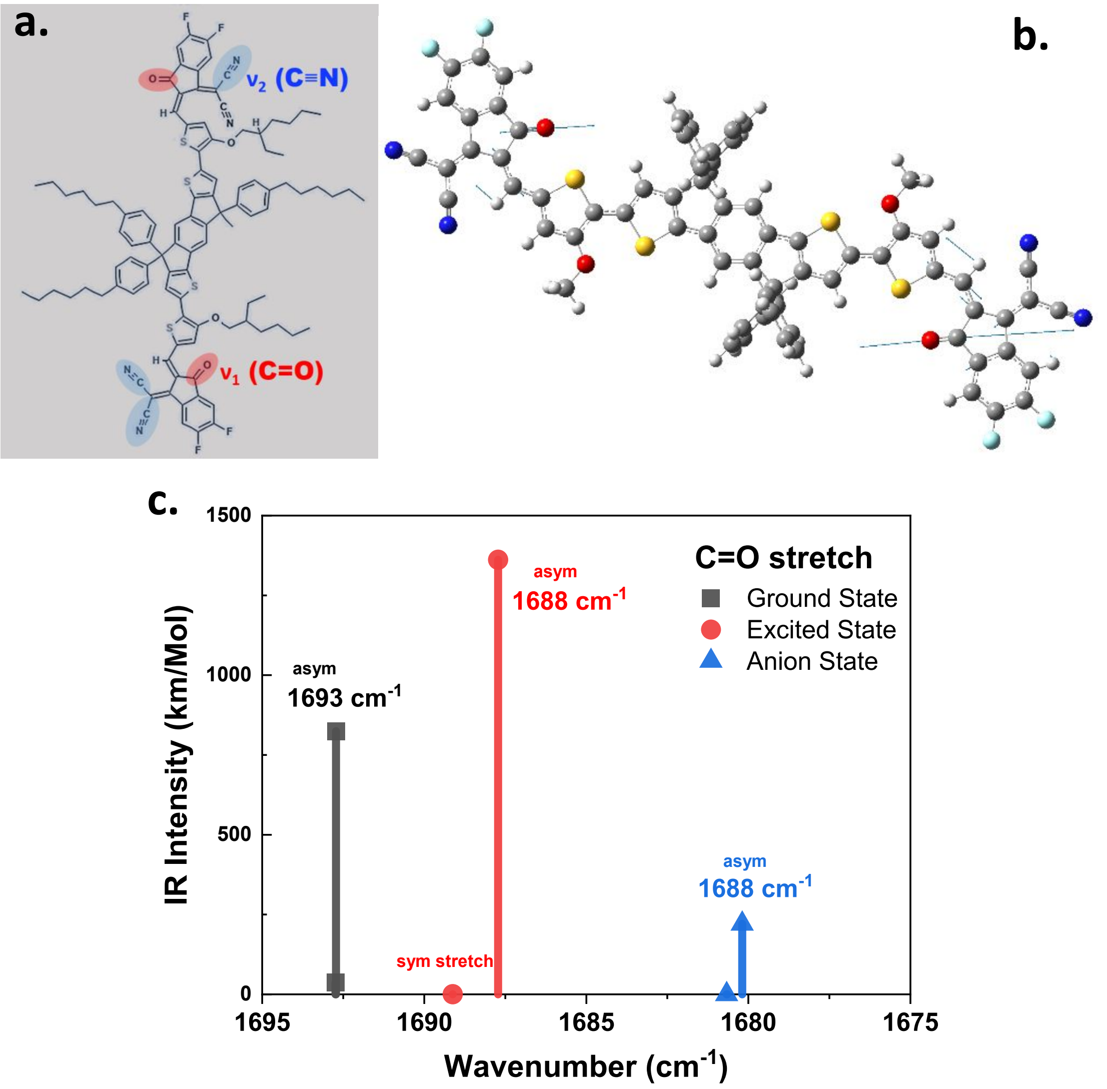

Figure S1. (a) The chemical structure of IEICO-4F molecule. (b) IEICO-4F molecule structure with the blue vectors corresponding to C=O stretching vibration. (c) The DFT calculated IR vibrational spectra of C=O vibrational stretch for the ground, excited and anionic states of IEICO-4F range from 1675 cm^{-1} to 1695 cm^{-1}, where 'asym' and 'sym' correspond to asymmetrical and symmetrical vibrational modes, respectively.

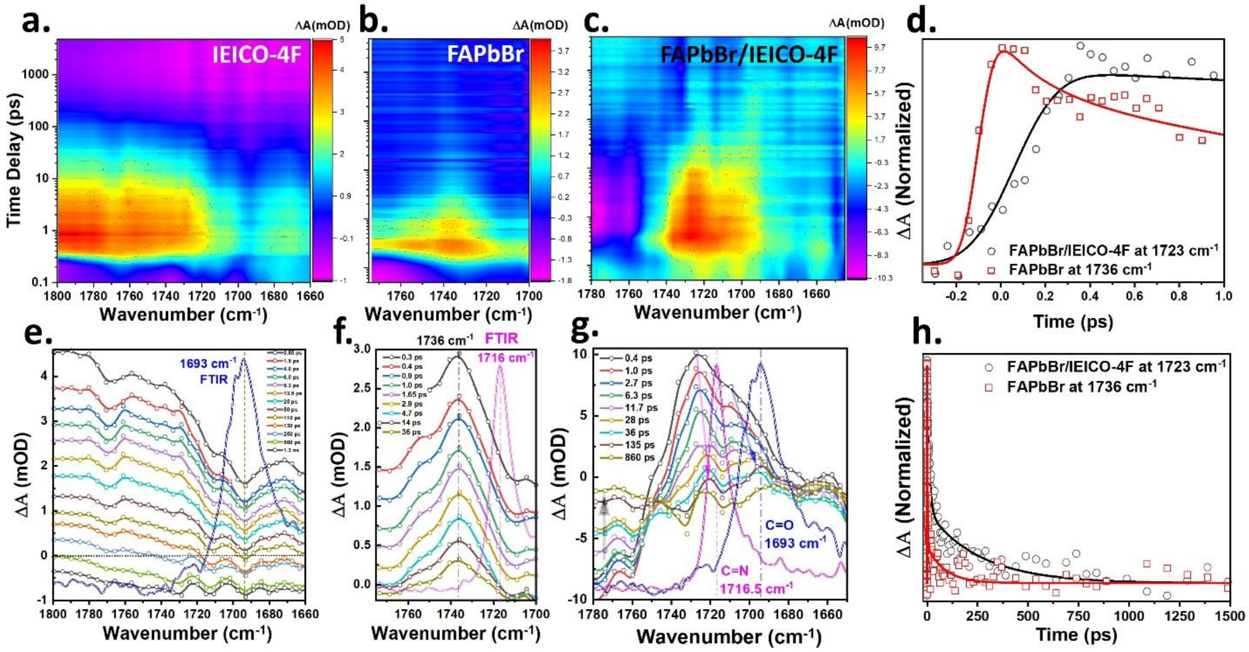

Figure S2. (a–c) The map plots of the of the fs-mid-IR spectroscopic measurements for IEICO-4F (C=O) after excitation at 750 nm, $FAPbBr_3$ (C=N) and mix of $FAPbBr_3$/IEICO-4F ν(C=O), ν(C=N) films after excitation at 450 nm respectively. (e–g) Their transient fs-mid-IR spectra respectively (inset: FT-IR at steady state, solid curve). (d) Kinetic plots of the transient mid-IR signal of ν(C=N) stretching vibration at 1723 cm^{-1} for $FAPbB_3$ film and ν(C=N) stretching vibration at 1736 cm^{-1} for $FAPbBr_3$/IEICO-4F in the range (d) from -0.25 to 1 ps, and (h) from -50 to 1500 ps.

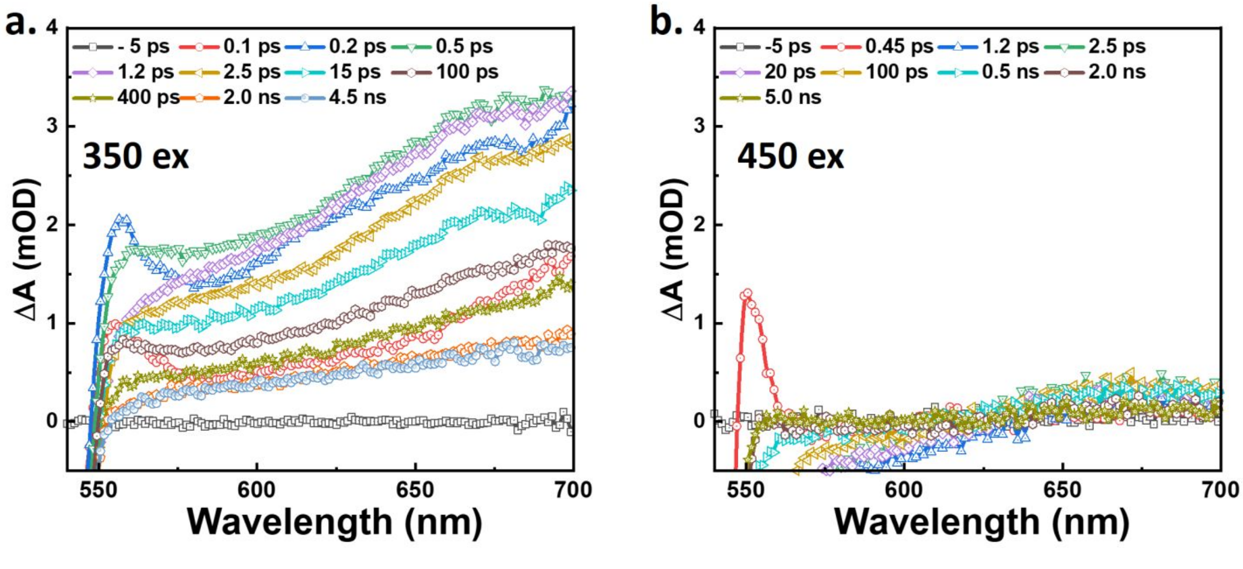

Figure S3. Spectra of fs-TA for $FAPbBr_3$ film under (a) 350 nm and (b) 450 nm excitation with fixed pump fluence 4 μJ/cm^2.

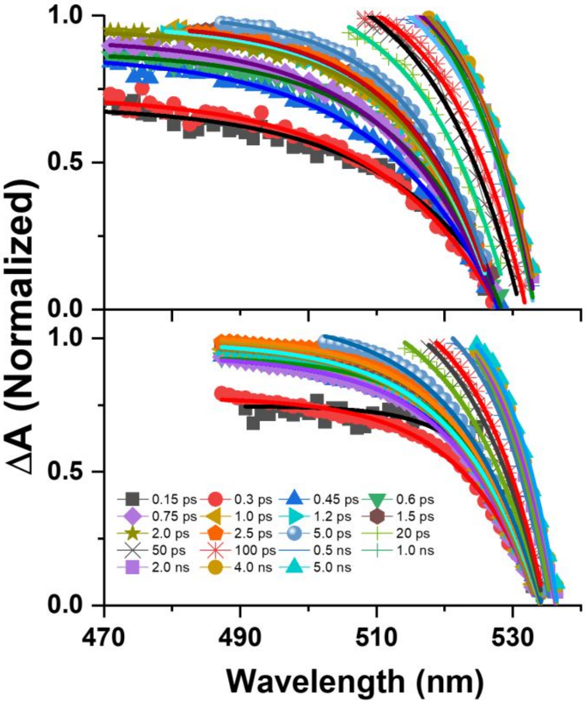

Figure S4. Single exponential fitting of high-energy tails in normalized fs-TA spectra of $FAPbBr_3$ and $FAPbBr_3$/IEICO-4F films after excitation at 350 nm with fixed pump fluence 4 μJ/cm^2.

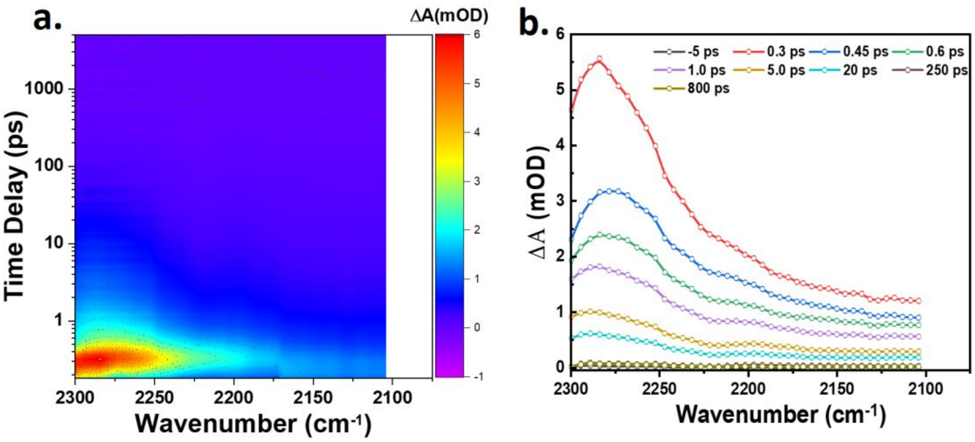

Figure S5. (a) The map plots of the mid-IR fs spectroscopic measurements for the IEICO-4F ν(C≡N) film after excitation at 730 nm and (b) the corresponding transient mid-IR spectra.

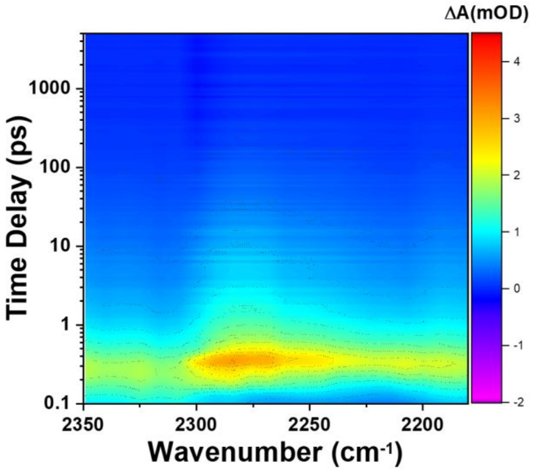

Figure S6. The map plots of the mid-IR fs spectroscopic measurements in the range 2180 – 2350 cm^{-1} for the IEICO-4F ν(C≡N) film after excitation at 450 nm .

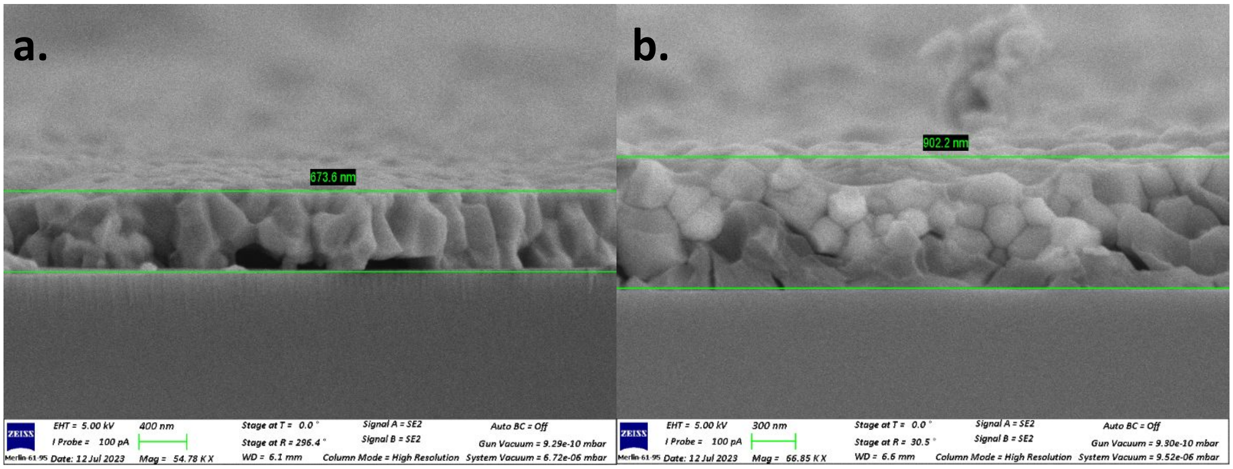

Figure S7. SEM images for (a) $FAPbBr_3$ and (b) mixture $FAPbBr_3$/IEICO-4F films.
