# Supplementary material for: Real-Time Tracking of Hot Carrier Injection at the Interface of FAPbBr3 Perovskite Using Femtosecond Mid-IR Spectroscopy
Source: ACS Cent Sci. 2023 Nov 3;10(1):43–53. doi: 10.1021/acscentsci.3c00562 (PMC10823510; doi:10.1021/acscentsci.3c00562)
Supplement: Supplementary file 2 — oc3c00562_si_002.pdf [file oc3c00562_si_002.pdf]

oc-2023-00562u.R1

Name: Peer Review Information for "Real-Time Tracking of Hot Carrier Injection at the Interface of FAPbBr3 Perovskite Using Femtosecond Mid-IR Spectroscopy"

First Round of Reviewer Comments

Reviewer: 1

Comments to the Author

The authors describe an interesting study of photogenerated electron injection from a perovskite thin film to a think film containg a near-IR absorbing dye using femtosecond transient absorption measurements. The main conclusion is that exciting the perovskite above its bandgap causes an electron to transfer to the dye layer. The title emphasizes mid-IR measurements, although the authors devote about the same amount of space to describing ultrafast experiments with probing at visible wavelengths. The phenomenon is interesting and important, but there are some concerns about the presentation and conclusions, which the authors must address in a revision.

Major issues

a. The authors assign a broad peak at 1693 cm-1 in IEICO-4F to C=O stretching, but the dye only has two carbonyl modes, so only 2 out of more than 100 carbon atoms are involved in C=O bonding but there are many more C=C bonds. It seems far more probable that the 1693 cm-1 mode is associated with ring stretching in the aromatic rings. The modes are likely to be strongly mixed. The authors write that the assignments were supported by DFT calculations, so they should provide the evidence for this. This calls into question the text on p. 3, line 42, left column.

b. Authors describe in the manuscript that formation of the two narrow positive peaks after 450 nm excitation, however those peaks are red shifted to lower frequency and broadened at 350 nm excitation. Peak shifts towards lower wavenumbers is explained by weakening of the C≡N bond due to heat produced by the generation of hot carriers at the interface. What is the evidence of higher heat production by the hot carriers (Tc) at 350 nm excitation? From Figure 2g it is not clear which Tc is extracted e.g whether it is from 450 nm or 350 nm excitation.

c. . How do the authors determine whether the mid-IR signal is due to electron transfer or due to excitation of IEICO-4F alone given the strong signals that they see when exciting just IEICO-4F at 450 nm? Could the red shift of the spectrum be explained by thermally induced change of the refractive index?

d. The authors did not discuss the thickness of the films or provide electronic microscopic images (SEM or TEM) of the FAPbBr3 perovskite. As the optical properties of lead halide perovskite depend upon the

size and shape of the material, it would be useful to see images that could correlate the size and shape with the optical properties.

e. A broad absorption of FAPbBr3 perovskite at wavelengths greater than 550 nm. However, according to previous reports, FAPbBr3 perovskite does not absorb here (J. Am. Chem. Soc. 2016, 138, 14202– 14205, J. Phys. Chem. Lett. 2020, 11, 4, 1239–1246). The authors should explain the discrepancy in their revised manuscript.

f. p. 6, lines 46-48, second column. The authors should specify more clearly there understanding of the electron donor and electron accetor. They write that electron injection originates with the phenol ring of the IEICO-4F dye, but inspection of the chemical structure of the dye shows that there is no phenol ring.

g. Given the strong cross secton of the C.triple bond.N mode, it is surprising that the mid-IR fs experments never show negative signals (bleaching). The authors should comment on why the bleaching signals are not seen. On p. 3, bands for C.triple bond.N stretching are seen at about 2216 cm-1 and about 2207 cm-1 (Figure 1d). Weakening of the CN bond upon electron transfer (anionic form) should result in red shifted bands, but the authors see blue shifts (Fig. 3 e and f) that furthermore depend on the excitation energy. This is very unclear and seems to undercut their conclusion that the CN anion is formed.

Minor issues

1. The author should show the chemical structure of IEICO-4F so the constituent atoms can be easily identified.

2. p. 2, Line 16 (“Here, both films exhibit…”). The preceding sentence describes three films, so the authors should describe what two (“both”) films are meant.

3. The authors should describe the thickness of the film layers.

4. p. 2, lines 27-28, right column. The authors should indicate where zero intensity is located on the graph in Figure 1C, so a reader can tell if the signal really decays to zero or not, or if an offset is present. It seems that there is no offset because the authors report lifetimes for a biexponential fit with amplitudes that sum to 100%. Also, in Figure 1C the individual markers should not be connected by lines. These lines create confusion near time zero for the red markers.

5. p. 2. The authors should replace “quadrupole” by “quadruple”.

6. Fig. 1D is drawn as if all of the FT-IR spectra have a common intensity axis, but the authors have likely scaled each segment by a different factor, obscuring the intensity differences between the various modes. This should be corrected and the authors should show the correct relative intensities for the various bands.

7. Figure 2D. Show the zero signal level.

8. The units are missing from the time axis in Fig. 3g.

9. The authors should describe the spot sizes and the fluence of the femtosecond laser pulses used in their measurements.

Reviewer: 2

Comments to the Author

In this paper, Nadinov et al. use multiple time-resolved spectroscopic techniques to monitor the injection of hot carriers at a perovskite-electron acceptor interface. While the overall premise of their experiments appears reasonable, their submission in its present form, needs improvement to justify publication, i.e. a major revision is necessary. The authors should address the following concerns:

1) What is the thickness of the FAPbBr3 and FAPbBr3/IEICO-4F films used in these studies? The authors mention shrinkage of the unit cell volume of FAPbBr3 due to incorporation of the charge acceptor. How are lattice parameters altered in the bulk, if as per their DFT calculations, structural relaxations mostly affect the interfacial region ?

2) While discussing the PB features in Fig. 2c on Page 3, the authors mention broadening and shift of the PB signals towards high-energy region. I believe, the shift towards longer wavelengths would imply less-energy.

3) Caption of Fig 2. d) and e) should be reserved. d) depicts shorter time window.

4) What is the instrument response function for their TA measurements?

5) Since the TOC figure indicates the relaxation pathways in terms of hot/cooled e- transfer, the authors may consider including the relevant timescales (< 150 fs, 205 fs) as well.

Author's Response to Peer Review Comments:

Omar F. Mohammed, PhD, FRSC, FIMMM
Associate Editor for ACS Applied Materials & Interfaces
Professor of Chemistry and Material Sciences and Engineering
Advanced Membranes & Porous Materials Center & KAUST Catalysis
Center Division of Physical Sciences and Engineering
Room 3277, Level 3, Building 5
KAUST, Thuwal 23955-6900, KSA
Room 3277, Level 3, Building 5
KAUST, Thuwal 23955-6900, KSA

Phone: +966 2 808 4491

July 18th, 2023

Thank you for your email on June 28th, 2023, regarding our manuscript oc-2023-00562u. We are grateful to the two referees for appreciating the importance of our work and approving it for publication in *ACS Central Science* after revision. In addition, we thank you so much for giving your attention to this work. Reviewers 1 and 2 raised some suggestions and concerns to improve the presentation of our manuscript, which we have fully addressed and clarified in the revised version of the manuscript in a point-by-point fashion, as you will see in the next paragraphs.

I hope that you will find our revised manuscript both compelling and publishable in *ACS Central Science*. I look forward to hearing from you soon.

Best regards,

Omar F. Mohammed, PhD, FRSC, IOP, FIMMM

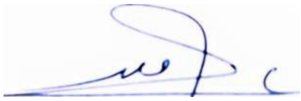

2019 & 2020 & 2021 & 2022 highly cited researcher (Web of Science)

Reviewer: 1

The authors describe an interesting study of photogenerated electron injection from a perovskite thin film to a think film containg a near-IR absorbing dye using femtosecond transient absorption measurements. The main conclusion is that exciting the perovskite above its bandgap causes an electron to transfer to the dye layer. The title emphasizes mid-IR measurements, although the authors devote about the same amount of space to describing ultrafast experiments with probing at visible wavelengths. The phenomenon is interesting and important, but there are some concerns about the presentation and conclusions, which the authors must address in a revision.

Response: We thank the reviewer for appreciating the importance of our work and for recommending publication in *ACS Central Science* after revision. The reviewer raised some concerns and suggestions to improve the presentation of our manuscript, which we have addressed in the revised version of the manuscript. Here is our response to the comments in a point-by-point fashion.

Major issues:

a. The authors assign a broad peak at 1693 cm-1 in IEICO-4F to C=O stretching, but the dye only has two carbonyl modes, so only 2 out of more than 100 carbon atoms are involved in C=O bonding but there are many more C=C bonds. It seems far more probable that the 1693 cm-1 mode is associated with ring stretching in the aromatic rings. The modes are likely to be strongly mixed. The authors write that the assignments were supported by DFT calculations, so they should provide the evidence for this. This calls into question the text on p. 3, line 42, left column.

Response: We appreciate the reviewer's comment. We agree that the size of the molecule will produce a complex FT-IR spectrum. However, the DFT and TD-DFT calculations support that the C=O absorption band appears isolated in the ground and excited states within a 100 cm^{-1} range, as shown in the Figures below. These bands correspond to the vibrational mode represented by the vectors in the structure below. As the reviewer kindly pointed out, the relative intensity of the C=O peak is small compared to other vibrational modes. To fully address this comment, we expanded the explanation on page 4 and included Figures S1(b-d) in the supporting information.

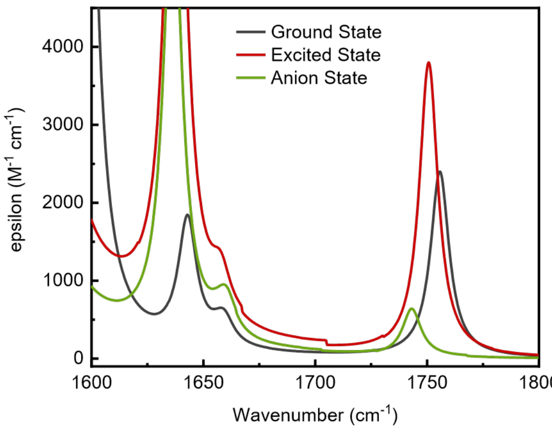

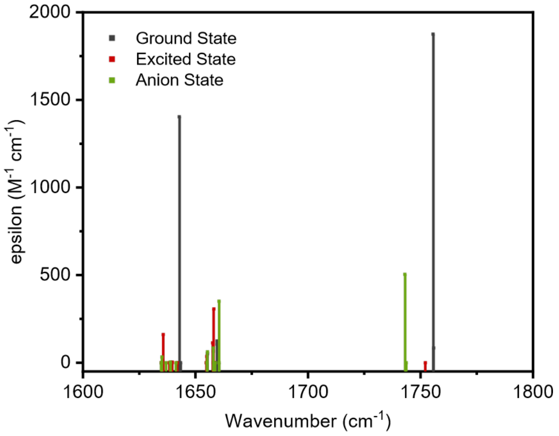

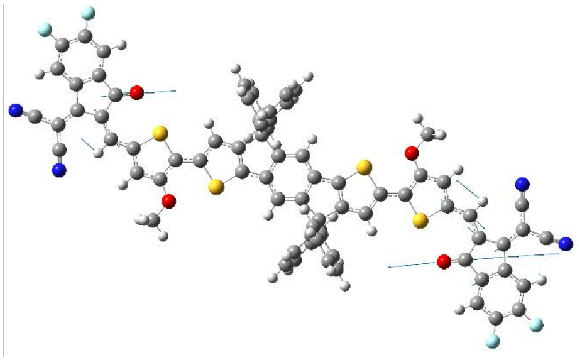

b. Authors describe in the manuscript that formation of the two narrow positive peaks after 450 nm excitation, however those peaks are red shifted to lower frequency and broadened at 350 nm excitation. Peak shifts towards lower wavenumbers is explained by weakening of the $C\equiv N$ bond due to heat produced by the generation of hot carriers at the interface. What is the evidence of higher heat production by the hot carriers (Tc) at 350 nm excitation? From Figure 2g it is not clear which Tc is extracted e.g whether it is from 450 nm or 350 nm excitation.

Response: We thank the reviewer for this remark. In our fs-TA measurements, we observed the generation of hot carriers upon 350-nm excitation in both pure $FAPbBr_3$ and mixed $FAPbBr_3$/IEICO-4F

films, as evident from the broadening and the shift in the PB signal at 538 nm (Figure 2c). These changes are attributed to the quasiequilibrium distribution of hot carriers in the perovskite crystal lattice and the bandgap renormalization process, respectively. In contrast, after 450 nm excitation for the same films, we did not observe any changes in the PB signal. Therefore, we believe that the weakening of the C≡N bond happens due to heat produced by the generation of hot carriers at the interface upon excess energy excitation at 350 nm. It should be noted that the excitation wavelength for $FAPbBr_3$ and $FAPbBr_3$/IEICO-4F films in Figure 2g was 350 nm. We have clarified this issue in the caption of Figure 2g (see page 4).

c. How do the authors determine whether the mid-IR signal is due to electron transfer or due to excitation of IEICO-4F alone given the strong signals that they see when exciting just IEICO-4F at 450 nm? Could the red shift of the spectrum be explained by thermally induced change of the refractive index?

Response: We thank the reviewer for pointing this out. In Figures 3d and e, we observe the spectral change of IEICO-4F with and without $FAPbBr_3$, representing neutral and anionic forms of IEICO-4F. The DFT calculation showed the charge redistribution in IEICO-4F after introducing the $FAPbBr_3$. In more detail, the anionic state exhibits a higher negative charge than the neutral state, which affects the weakening of the C≡N bond character

(Figure 4c). The observed shift of the C≡N in our measurement was predicted by the DFT calculation (Figure 4d). To ensure that the change in the refractive index does not cause the shift, we repeated the same experiments for different thicknesses, and the results were the same.

d. The authors did not discuss the thickness of the films or provide electronic microscopic images (SEM or TEM) of the FAPbBr3 perovskite. As the optical properties of lead halide perovskite depend upon the size and shape of the material, it would be useful to see images that could correlate the size and shape with the optical properties.

Response: We thank the reviewer for pointing this out. The film thicknesses were determined by two methods. The first method was using the Tencor stylus profiler surface measurement system (Tencor P-6) by applying a 0.5 mN force on the probing tip, where the film thickness is typically measured across a scratch. The average value of the thicknesses after measurements at different areas with the profiler is 610 nm for pure $FAPbBr_3$ and 1070 nm for mixture $FAPbBr_3$/IEICO-4F. The second method of thickness determination is the cross-sectional SEM images of $FAPbBr_3$ and mixture $FAPbBr_3$/IEICO-4F. As is seen in the figures below, the thicknesses of the samples with SEM (673 nm for pure $FAPbBr_3$, and 902 nm for mixture $FAPbBr_3$/IEICO-4F) are in close agreement with the thickness measurements we found with the profiler. In the revised version of the manuscript, we expanded the discussion about the thicknesses of the samples in the Experimental Methods section – film fabrication (on page 9) to clarify this point and included the SEM images in Figure S5 in the supporting information.

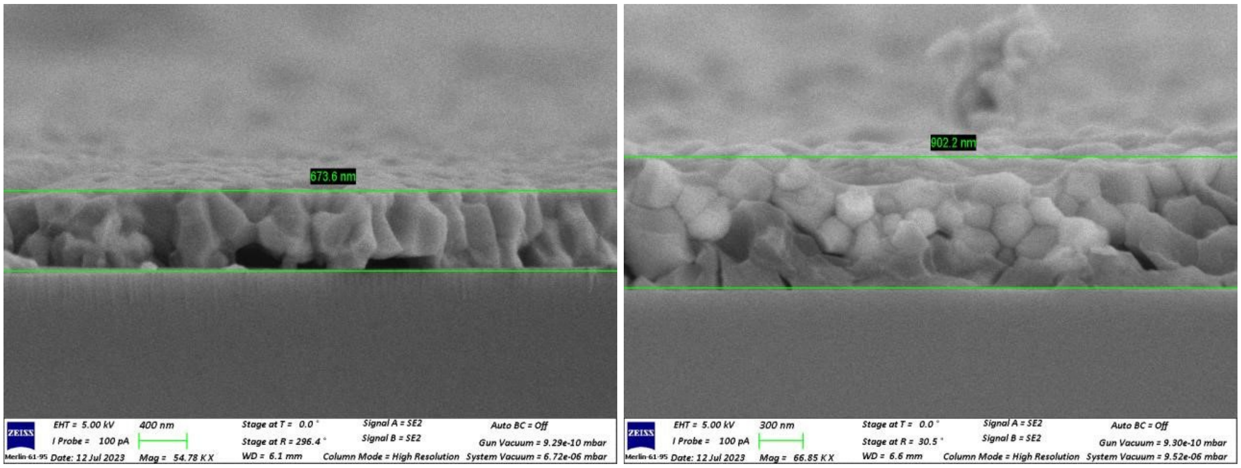

e. A broad absorption of FAPbBr3 perovskite at wavelengths greater than 550 nm. However, according to previous reports, FAPbBr3 perovskite does not absorb here (J. Am. Chem. Soc. 2016, 138, 14202– 14205, J. Phys. Chem. Lett. 2020, 11, 4, 1239–1246). The authors should explain the discrepancy in their revised manuscript.

Response: We thank the reviewer for pointing this out. Our absorption measurements were performed on spincoated films. The difference between nanocrystals in colloidal solution and solid-state materials can cause the appearance of surface defects that can lead to a tail in the absorption band of the $FAPbBr_3$. This feature has been found in other $FAPbBr_3$ films, and this strong band-tail (535–800 nm) has been associated to electron–phonon coupling due to the bound carriers below the band edge.[1, 2] We expanded the discussion on page 3 to clarify this issue and added the references 47 and 48.

- Doumbia, Y.; Bouich, A.; Mari Soucase, B.; Soro, D. *Opt. Mater.* **2023**, *135*, 113321.
- Guo, Y.; Zou, B.; Yang, F.; Zheng, X.; Peng, H.; Wang, J. *Phys. Chem. Chem. Phys.* **2021**, *23*, 10153-10163.

f. p. 6, lines 46-48, second column. The authors should specify more clearly there understanding of the electron donor and electron accetor. They write that electron injection originates with the phenol ring of the IEICO-4F dye, but inspection of the chemical structure of the dye shows that there is no phenol ring.

Response: We thank the reviewer for this remark. The reviewer was correct, it was a typo in the draft, and we replaced the "phenol ring" with " alkyl substituted phenyl ring" on page 7.

g. Given the strong cross secton of the C.triple bond.N mode, it is surprising that the mid-IR fs experments never show negative signals (bleaching). The authors should comment on why the bleaching signals are not seen. On p. 3, bands for C.triple bond.N stretching are seen at about 2216 cm-1 and about 2207 cm-1 (Figure 1d). Weakening of the CN bond upon electron transfer (anionic form) should result in red shifted bands, but the authors see blue shifts (Fig. 3 e and f) that furthermore depend on the excitation energy. This is very unclear and seems to undercut their conclusion that the CN anion is formed.

Response: We thank the reviewer for pointing this out. The reviewer is correct in mentioning that we do not see the clear bleach signal of the $C\equiv N$ band, and the reason is the strong/intense positive signal of

the C≡N band overlapped with the bleach signal. Interestingly we start to observe the signature of the bleach signal after 1 ps at 2215 cm^{-1} (Figure 3d) when the intensity of the positive signal starts to decay. In Figure 3d we observe transient mid-IR spectra for the pure IEICO-4F under 450 nm excitation assigned to IEICO-4F neutral form. Here we observe a single broad peak at 2276 cm^{-1}. The blue shift is observed for IEICO4F neutral form in the excited state. On the other hand, for the mix $FAPbBr_3$/IEICO-4F under 450 nm excitation, we form an anionic IEICO-4F state. In this case, we start to observe clearly two peaks, the intense one at 2233 cm^{-1} and the less intense one at 2115 cm^{-1}. This means that peaks shifted to a lower wavenumber (red shifted), which is attributed to the weakening of the C≡N band compared to the neutral form. In Figures 3e and f, we compare the same sample $FAPbBr_3$/IEICO-4F (in anionic form), but under different excitation wavelengths where we see the effect of hot electrons on the spectrum. We have expanded the discussion on page 6 to clarify these issues.

Minor issues:

1. The author should show the chemical structure of IEICO-4F so the constituent atoms can be easily identified.

Response: We thank the reviewer for this remark. As requested, we added the chemical structure of IEICO-4F in the supporting information in Figure S1 a.

2. p. 2, Line 16 (“Here, both films exhibit…”). The preceding sentence describes three films, so the authors should describe what two (“both”) films are meant.

Response: We thank the reviewer for pointing this out. We modified the sentence by naming the samples (please see page 2).

3. The authors should describe the thickness of the film layers.

Response: We thank the reviewer for this remark. We have addressed this question in the comment e. Therefore, we expanded the discussion about the thicknesses of the samples in the Experimental Methods section – film fabrication (please see page 9).

4. p. 2, lines 27-28, right column. The authors should indicate where zero intensity is located on the graph in Figure 1C, so a reader can tell if the signal really decays to zero or not, or if an offset is present. It seems that there is no offset because the authors report lifetimes for a biexponential fit with amplitudes that sum to 100%. Also, in Figure 1C the individual markers should not be connected by lines. These lines create confusion near time zero for the red markers.

Response: We thank the reviewer for this remark. In the updated version of Figure 1c, we showed the zero level in the Y axis (Intensity) and removed the individual markers as kindly suggested.

5. p. 2. The authors should replace “quadrupole” by “quadruple”.

Response: We thank the reviewer for this remark. We corrected the typo in the revised version (page 4).

6. Fig. 1D is drawn as if all of the FT-IR spectra have a common intensity axis, but the authors have likely scaled each segment by a different factor, obscuring the intensity differences between the various

modes. This should be corrected and the authors should show the correct relative intensities for the various bands.

Response: We thank the reviewer for pointing this out. We add the magnification value for the Y axis in the updated version (IR Intensity in Figure 1d) for each spectrum, making it easier to compare their intensities. We just want to show unchanged vibrational peak positions after mixing, especially for the CN band of IEICO-4F before and after adding the $FAPbBr_3$.

7. Figure 2D. Show the zero signal level.

Response: We thank the reviewer for this remark. As kindly suggested, we show the zero level in the revised version of Figure 2 d

8. The units are missing from the time axis in Fig. 3g.

Response: We thank the reviewer for pointing this out. Figure 3 g and h have the same unit as Time (ps). It is better to keep it in this format.

9. The authors should describe the spot sizes and the fluence of the femtosecond laser pulses used in theirmeasurements.

Response: We thank the reviewer for this remark. We expanded the discussion on page 9 about the excitation spot size and the fluence in the Experimental Methods section – Time-Resolved Spectroscopy.

Reviewer: 2

In this paper, Nadinov et al. use multiple time-resolved spectroscopic techniques to monitor the injection of hot carriers at a perovskite-electron acceptor interface. While the overall premise of their experiments appears reasonable, their submission in its present form, needs improvement to justify publication, i.e. a major revision is necessary. The authors should address the following concerns:

Response: We thank the reviewer for appreciating the importance of our work and for recommending publication in *ACS Central Science* after revision. Reviewer 2 raised some minor concerns for clarification to improve the presentation of our manuscript, and we have addressed these concerns in the revised version of the manuscript. Here is our response to the reviewer's comments in a point-by-point fashion.

1. What is the thickness of the FAPbBr3 and FAPbBr3/IEICO-4F films used in these studies? The authors mention shrinkage of the unit cell volume of FAPbBr3 due to incorporation of the charge acceptor. How are lattice parameters altered in the bulk, if as per their DFT calculations, structural relaxations mostly affect the interfacial region ?

Response: We thank the reviewer for pointing this out. The thicknesses of $FAPbBr_3$ and $FAPbBr_3$/IEICO-4F films are around 640 nm and 980 nm, respectively. In the revised version of the manuscript, we add information about the thicknesses of the samples in the Experimental Methods section – film fabrication (page 9). According to the DFT calculation, the Pb-Br distances in $FAPbBr_3$ expand and shrink at the interface, and the bond length varies between 2.88-3.2 Å compared to the bulk value of 3.00 Å. The bond length change mainly happens on the first layer of the $FAPbBr_3$. For the inner layers, the bond

length variation is marginal. Additionally, from Figure 4a, we clearly see that only the first layer of $FAPbBr_3$ is involved in the charge transfer. Therefore, we can conclude that the change in the bond length in the system is confined to the interfacial region, as indicated and clarified in the revised version of the manuscript.

2. While discussing the PB features in Fig. 2c on Page 3, the authors mention broadening and shift of the PB signals towards high-energy region. I believe, the shift towards longer wavelengths would imply less-energy.

Response: We thank the reviewer for pointing this out. We agree with the reviewer that we observed the shift of the PB feature from high energy to lower energy after excitation, so we corrected this sentence (please see Page 5).

3. Caption of Fig 2. d) and e) should be reserved. d) depicts shorter time window.

Response: We thank the reviewer for this remark. The order in the caption for Figures 2d and e has been corrected.

4. What is the instrument response function for their TA measurements?

Response: We thank the reviewer for pointing this out. The IRF for the TA measurements is 168 fs which was obtained by measuring the scattering of methanol and is shown in the figure below.

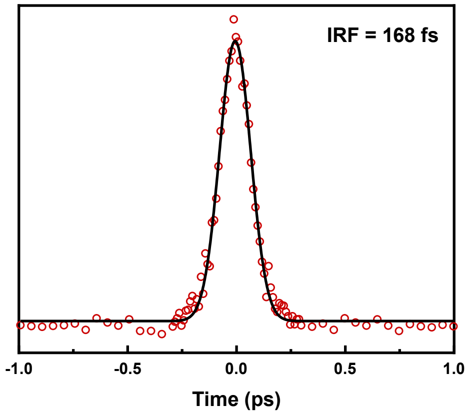

5. Since the TOC figure indicates the relaxation pathways in terms of hot/cooled e- transfer, the authors may consider including the relevant timescales (< 150 fs, 205 fs) as well.

Response: We thank the reviewer for the good recommendation. As suggested, we included the timescale for the hot/cooled e- transfer in the TOC figure.

oc-2023-00562u.R2

Name: Peer Review Information for "Real-Time Tracking of Hot Carrier Injection at the Interface of FAPbBr3 Perovskite Using Femtosecond Mid-IR Spectroscopy"

Second Round of Reviewer Comments

Reviewer: 1

Comments to the Author

The authors made many improvements to the article, but the IR spectroscopy discussion remains a weak point, and, unfortunately, some of the IR spectroscopy parts that were added (Figure S1) have errors. Because the authors have chosen to highlight the fs mid-IR spectroscopy with their title (even though their conclusions rest also on other techniques as pointed out in my first review), it only makes sense to this reviewer for the authors to “get it right”. The paper should thus undergo a further round of revision because of concerns about the interpretation of the fs mid-IR spectroscopy.

Their responses to my points a, c, and g are problematic (the other points have been addressed satisfactorily):

a. The figures that the authors provided in response to my question about the assignment of the experimental band at 1693 cm-1, and which are shown now in Figure S1, are difficult to interpret and appear to have errors. Figure S1(d) presumably shows stick spectra that graph oscillator strength vs. frequency. The vertical axis label, however, is labeled as molar absorption coefficient (extinction coefficient). Oscillator strength is proportional to the integral of the lineshape, so the very narrow (“sticks”) lines shown in Figure S1(d) should have equal area as the broader bands shown in Figure S1(c), but they cannot according to the vertical axis units shown in each graph. I suspect that the authors have simply drawn lines in Figure S1(d) that are equal in height to the oscillator strength of the transitions and incorrectly labeled the vertical axis as extinction.

An even more serious problem is that the graph in Figure S1(c) does not agree with what is shown in Figure S1(d). Using the excited state (red) as an example, the band near 1650 cm-1 in Figure S1(c) is much stronger than the band for the ground state (black), but the opposite trend is seen for the oscillator strengths in Figure S1(d). A similar observation holds for the band near 1750 cm-1. This band is greatest for the excited state (red) in Figure S1(c) even though it has the lowest oscillator strength of all states simulated in Figure S1(d). This does not appear to be a problem of switching the colors as this

reviewer can see no way to make what is shown in panel d agree with what is shown in panel c. It must also be noted that the authors fail to describe in the Computational Methods section how the broadened lines shown in Figure S1 and in main text Figure 4d were generated. There is also no description anywhere of what scaling factor was used to compare DFT-estimated frequencies with experimental ones. This information must be included.

Returning to the authors' reply to my question about the 1693 cm-1 experimental band, their response and Figure S1 are insufficient. They illustrate nuclear displacements for the C=O stretching mode, but the authors do not identify the corresponding line in their spectra in FigureS1c,d. Many mode frequencies are seen in two clusters of lines between 1600 and 1800 cm-1. Which one corresponds to the C=O stretching frequency that they have illustrated? If it is the higher frequency band near 1750 cm-1 (note my comment above about the authors' failure to indicate whether computed vibrational spectra were scaled in any way), then what is the assignment of the very strong bands seen in Figure S1c near 1650 cm-1? There appears to be a small peak above 1720 cm-1 in the FTIR spectrum of IEICO-4F shown in Figure 1d. The authors should consider whether this is the weak C=O band, and they should show the entire FTIR spectra of the samples shown in Figure 1, so they can be properly assessed.

Their statement that they "expanded the explanation on p. 4" is not supported by an examination of the revised Word document with yellow highlighting, which shows merely the addition of the phrase "See Figure S1b-d".

c. The authors misunderstood my earlier comment. I am pointing out that the IEICO-4F film shows almost no absorption at 450 nm, according to Figure 1b. If this is the case, then how is it that they observe such strong signals when they excite the bare film at 450 nm (Fig. 3d)? The authors should state what the absorbance of the film is at their pump wavelength for Fig. 3d. Did they verify that the signal changes linearly with changes to the pump intensity? The very broad character of the spectrum raises a question of whether this is a kind of multiphoton effect as mid-IR spectrum looks extremely unusual (but it looks like IRAV, see below). They should present transient mid-IR data on the film excited at 800 nm and compare this with what is shown in Fig. 3d to be sure that they are really capturing excitons in the film.

g. The authors' explanation that they observe the bleach after 1 ps when the excited state absorption begins to decrease is not credible as the IR difference spectrum is determined at all times (not just at later delay times) by the difference spectrum between the excited state and the ground state. Also, the authors did not explain how the nitrile stretch frequency of the anionic form could be blue shifted relative to the ground state nitrile stretch frequency if the nitrile bond has weakened. This reviewer knows of no precedent for a nitrile stretch frequency in the excited state that is blue shifted relative to the ground state frequency. Is this predicted by their calculations? The authors should add their calculated spectrum for the ground state to Figure 4d, so the reader can evaluate this.

The weak bleach feature at 2215 cm-1 in Figure 3d is not visible in most of the transient spectra shown. This is a strong mode in the ground state. How can this mode be so much stronger in the excited state (authors' revised statement on p. 6)? The only precedent that this reviewer knows is in donor-acceptor systems that show IRAV (so-called infrared active vibrations) bands. See papers [1-2]. Actually, the IEICO-4F acceptor is a good candidate for IRAV bands based on its A-D-A character, but as I noted above, the authors excite it at a wavelength where it seems to have no absorbance, so they must provide evidence supporting why they believe they are forming excited states in this material. The very broad mid-IR spectra shown in Figure 3 suggest that these are electronic transitions or at least vibrations that are heavily mixed with electronic transitions.

[1] B. Dereka et al., Excited-State Symmetry Breaking in a Quadrupolar Molecule Visualized in Time and Space. J. Phys. Chem. Lett. 2017, 8 (24), 6029–6034. https://doi.org/10.1021/acs.jpclett.7b02944.

[2] W. J. Kendrick et al., Mechanisms of IR Amplification in Radical Cation Polarons. Chem. Sci. 2020, 11 (8), 2112–2120. https://doi.org/10.1039/C9SC05717J.

My careful review of the authors' responses and the mid-IR spectroscopy that is presented has raised several additional questions/concerns that the reviewers should also address:

1. The statement that the authors make on p. 2 is problematic:

"The calculations reveal that electron injection weakens the C≡N bond character in the acceptor material". The authors do not indicate clearly which band in Figure 3e and 3f is the symmetric and which is the asymmetric stretch. Regardless, one of these bands is higher in frequency than either band in the ground electronic state of the neutral (Figure 3d). How can the C≡N be weaker if one of its modes is blue shifted to a higher frequency? The authors must justify this carefully, and they must show more of the assignments from their DFT calculations, also for anions, and show where the symmetric and asymmetric stretches are predicted to occur.

All mid-IR fs spectra in Figure 3(d-f) show a sharp drop near 2300 cm-1. Have the authors considered whether they are accurately able to measure these signals, which fall near the edge of their detection window? They should test by re-centering their mid-IR probe pulse at 2300 cm-1 and comparing transient spectra measured there with the ones shown in the paper.

2. The DFT calculations show a tremendous difference in intensity of the symmetric vs. asymmetric stretching modes of the nitrile group (Fig. 4d). This is not matched by the more similar amplitudes in Fig. 3e,f. Also, the splitting is ~100 cm-1 in the experiments, but only ~10-20 cm-1 in the calculations. In agreement with the calculations, there is a small ~10 cm-1 splitting for the ground state of IEICO-4F and a very weak shoulder (Fig. 1d). The authors' calculations in Figure 4d reproduce neither the experimentally observed splitting nor the amplitudes seen in the experimental spectra. If so little about the calculated spectrum matches experiment, then how can the authors' assignment be justified? The authors must address this.

3. Can the authors more clearly explain how ns PL lifetimes are observed even though charge injection takes place on a sub-ps timescale? A clear explanation is warranted for the general readership of ACS Central Science.

Some minor issues remain:

M1. In Figure 1d the authors illustrate a vertical scale multiplier for the middle portion of the FTIR spectra, but the number in front of the 'x' character is missing for the black curve. If the nitrile stretch is unchanged, then is the scaling 3.5 x as in the red spectrum?

M2. Figure 4c. The authors do not state what quantity is shown by the color bars and what the units are.

M3. Reviewer 2 is correct that the authors should state their IRF in the paper. The authors communicated it to the reviewers, but it should be in the

Author's Response to Peer Review Comments:

KING ABDULLAH UNIVERSITY OF SCIENCE AND TECHNOLOGY
Division of Physical Sciences and Engineering, Thuwal 23955-6900, KSA

Omar F. Mohammed, PhD, FRSC, FIMMM
Associate Editor for ACS Applied Materials & Interfaces
Professor of Chemistry and Material Sciences and Engineering
Advanced Membranes & Porous Materials Center & KAUST Catalysis Center
Division of Physical Sciences and Engineering
Room 3277, Level 3, Building 5

KAUST, Thuwal 23955-6900, KSA Room
3277, Level 3, Building 5
KAUST, Thuwal 23955-6900, KSA
Phone: +966 2 808 4491

September 8th, 2023

Thank you for your email on August 24th, 2023, regarding our manuscript oc-2023-00562u.R1. We are grateful again to the referee for recommending publication in *ACS Central Science* after revision. In addition, we thank you so much for giving your attention to this work. The reviewer raised a few additional suggestions and concerns to further improve the presentation and clarity of our manuscript, which we have fully addressed in the revised version of the manuscript. You will see in the next paragraphs our response to the reviewers' comments in a point-by-point fashion.

Finally, I hope that you will find our revised manuscript both compelling and publishable in *ACS Central Science*. I look forward to hearing from you soon.

Best regards,

Omar F. Mohammed, PhD, FRSC, IOP, FIMMM

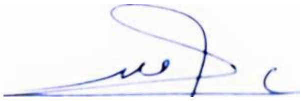

2019 & 2020 & 2021 & 2022 highly cited researcher (Web of Science)
Associate Editor for ACS Applied Materials & Interfaces

Reviewer: 1

The authors made many improvements to the article, but the IR spectroscopy discussion remains a weak point, and, unfortunately, some of the IR spectroscopy parts that were added (Figure S1) have errors. Because the authors have chosen to highlight the fs mid-IR spectroscopy with their title (even though their conclusions rest also on other techniques as pointed out in my first review), it only makes sense to this reviewer for the authors to "get it right". The paper should thus undergo a further round of revision because of concerns about the interpretation of the fs mid-IR spectroscopy.

Their responses to my points a, c, and g are problematic (the other points have been addressed satisfactorily):

Response: We thank the reviewer for appreciating the effort made to address most of the concerns made earlier by the reviewers. We also thank the reviewer for the additional concerns and suggestions to further improve the presentation of our manuscript. We have addressed these concerns in the revised version of the manuscript. Here is our response to the comments in a point-by-point fashion.

Major issues:

a. The figures that the authors provided in response to my question about the assignment of the experimental band at 1693 cm-1, and which are shown now in Figure S1, are difficult to interpret and appear to have errors. Figure S1(d) presumably shows stick spectra that graph oscillator strength vs. frequency. The vertical axis label, however, is labeled as molar absorption coefficient (extinction coefficient). Oscillator strength is proportional to the integral of the lineshape, so the very narrow ("sticks") lines shown in Figure S1(d) should have equal area as the broader bands shown in Figure S1(c), but they cannot according to the vertical axis units shown in each graph. I suspect that the authors have simply drawn lines in Figure S1(d) that are equal in height to the oscillator strength of the transitions and incorrectly labeled the vertical axis as extinction.

An even more serious problem is that the graph in Figure S1(c) does not agree with what is shown in Figure S1(d). Using the excited state (red) as an example, the band near 1650 cm-1 in Figure S1(c) is much stronger than the band for the ground state (black), but the opposite trend is seen for the oscillator strengths in Figure S1(d). A similar observation holds for the band near 1750 cm-1. This band is greatest for the excited state (red) in Figure S1(c) even though it has the lowest oscillator strength of all states simulated in Figure S1(d). This does not appear to be a problem of switching the colors as this reviewer can see no way to make what is shown in panel d agree with what is shown in panel c. It must also be noted that the authors fail to describe in the Computational Methods section how the broadened lines shown in Figure S1 and in main text Figure 4d were generated. There is also no description anywhere of what scaling factor was used to compare DFT-estimated frequencies with experimental ones. This information must be included.

Returning to the authors' reply to my question about the 1693 cm-1 experimental band, their response and Figure S1 are insufficient. They illustrate nuclear displacements for the C=O stretching mode, but the authors do not identify the corresponding line in their spectra in FigureS1c,d. Many mode frequencies are seen in two clusters of lines between 1600 and 1800 cm-1. Which one corresponds to the C=O stretching frequency that they have illustrated? If it is the higher frequency band near 1750 cm-1 (note my comment above about the authors' failure to indicate whether computed vibrational spectra were scaled in any way), then what is the assignment of the very strong bands seen in Figure S1c near 1650 cm-1? There appears to be a small peak above 1720 cm-1 in the FTIR spectrum of IEICO-4F shown in Figure 1d. The authors should consider whether this is the weak C=O band, and they should show the entire FTIR spectra of the samples shown in Figure 1, so they can be properly assessed.

Their statement that they "expanded the explanation on p. 4" is not supported by an examination of the revised Word document with yellow highlighting, which shows merely the addition of the phrase "See Figure S1b-d".

Response: We sincerely appreciate the reviewer's insightful comment which brought to light the errors within Figure S1(c and d), that we fully acknowledge. To address these concerns and avoid any possible future confusion, we have made a major revision and significant modification to the figure. We have transitioned the figure from the initial arrangement to a singular graph, designed to illustrate IR intensity (km/Mol) as a function of wavenumber (cm^{-1}), specifically in the range of 1675 cm^{-1} to 1695 cm^{-1}. Our revised version highlights the C=O vibrational band in the ground, excited, and anionic states, clearly distinguishing between symmetrical and asymmetrical stretches. We have also adjusted the peak position for better alignment with the core data.

Considering the reviewer's valuable feedback about the existence of broadened lines in Figure S1c, we responded to this to exclude these elements. Being in this regime, we agree with the reviewer in saying that the DFT calculation result includes only the intensities and positions of IR vibrational modes. Also, we agree that the IR peak Half-Width at Half Height parameter is merely a manually-set factor and does not contribute additional data about the calculation. Consequently, we have fixed the issue with the broadening in Figure 4d. To further address the reviewer's concerns on our explanation of DFT calculations related to the C=O stretching mode, we have expanded on this topic on page 3 of the revised version of the manuscript. Finally, we have now placed the corrected Figure S1c within the supporting information.

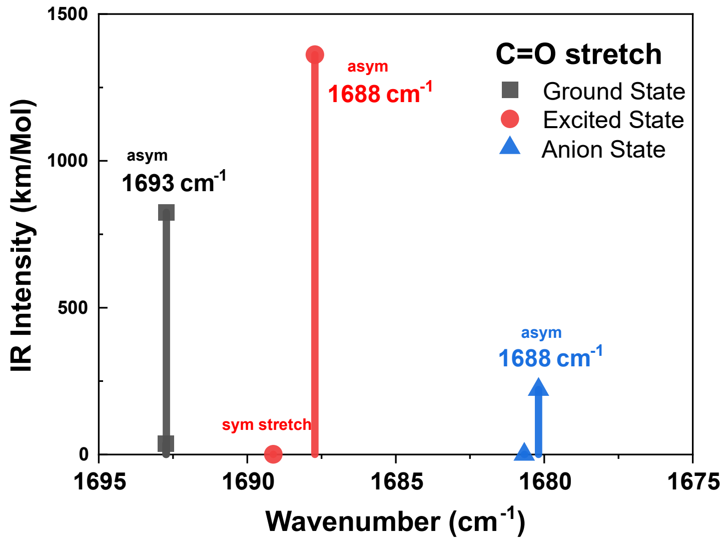

c. The authors misunderstood my earlier comment. I am pointing out that the IEICO-4F film shows almost no absorption at 450 nm, according to Figure 1b. If this is the case, then how is it that they observe such strong signals when they excite the bare film at 450 nm (Fig. 3d)? The authors should state what the absorbance of the film is at their pump wavelength for Fig. 3d. Did they verify that the signal changes linearly with changes to the pump intensity? The very broad character of the spectrum raises a question of whether this is a kind of multiphoton effect as mid-IR spectrum looks extremely unusual (but it looks like IRAV, see below). They should present transient mid-IR data on the film excited at 800 nm and compare this with what is shown in Fig. 3d to be sure that they are really capturing excitons in the film.

Response: We thank the reviewer for the remark. As a matter of fact, the absorption kindly mentioned by the reviewer at 450 nm is notably lower than the absorption within the range of 700 nm to 900 nm; however, it is not negligible. As kindly suggested by the reviewer, we have conducted supplementary transient mid-IR experiments on IEICO-4F film, employing an excitation wavelength of 730 nm (as depicted below, in comparison with Figures 3a and 3d). From these experiments, we ascertain a similar spectrum and shape of the

C≡N vibrational band. This serves as evidence to exclude any kind of multiphoton effects upon 450-nm excitation.

In order to comprehensively address this question, we have expanded the discussion in the 'Mid-IR fs Spectroscopy for $FAPbBr_3$ and $FAPbBr_3$/IEICO-4F' section on page 6 of the revised manuscript. Additionally, we have included the relevant figure (IEICO-4F under 730 nm excitation) as Figure S5 in the supporting information.

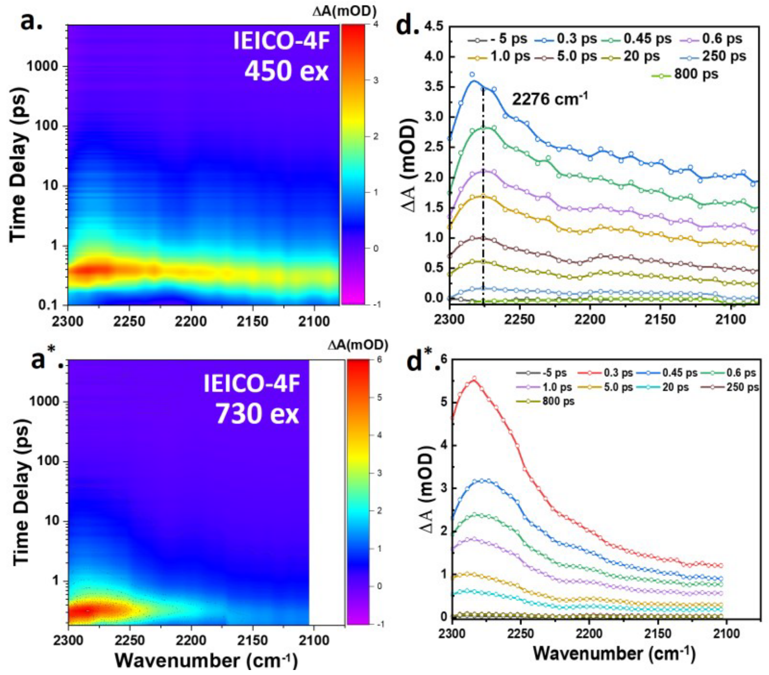

g. The authors' explanation that they observe the bleach after 1 ps when the excited state absorption begins to decrease is not credible as the IR difference spectrum is determined at all times (not just at later delay times) by the difference spectrum between the excited state and the ground state. Also, the authors did not explain how the nitrile stretch frequency of the anionic form could be blue shifted relative to the ground state nitrile stretch frequency if the nitrile bond has weakened. This reviewer knows of no precedent for a nitrile stretch frequency in the excited state that is blue shifted relative to the ground state frequency. Is this predicted by their calculations? The authors should add their calculated spectrum for the ground state to Figure 4d, so the reader can evaluate this.

The weak bleach feature at 2215 cm-1 in Figure 3d is not visible in most of the transient spectra shown. This is a strong mode in the ground state. How can this mode be so much stronger in the excited state (authors' revised statement on p. 6)? The only precedent that this reviewer knows is in donor-acceptor

systems that show IRAV (so-called infrared active vibrations) bands. See papers [1-2]. Actually, the IEICO-4F acceptor is a good candidate for IRAV bands based on its A-D-A character, but as I noted above, the authors excite it at a wavelength where it seems to have no absorbance, so they must provide evidence supporting why they believe they are forming excited states in this material. The very broad mid-IR spectra shown in Figure 3 suggest that these are electronic transitions or at least vibrations that are heavily mixed with electronic transitions.

[1] B. Dereka et al., Excited-State Symmetry Breaking in a Quadrupolar Molecule Visualized in Time and Space. J. Phys. Chem. Lett. 2017, 8 (24), 6029–6034.
[2] W. J. Kendrick et al., Mechanisms of IR Amplification in Radical Cation Polarons. Chem. Sci. 2020, 11 (8), 2112–2120.

Response: We thank the reviewer very much for this remark. As the reviewer knows, a blue shift in vibrational bands denotes an increase in the vibrational frequency. This phenomenon can emerge from changes in bond strength or/and bond length due to alterations in the electronic or structural characteristics of the molecule upon excitation including charge delocalization. However, the precise outcome hinges on the molecular system and the attributes of the excited state.

In our manuscript, we exclusively compare the positions of the CN vibrational band (both symmetrical and asymmetrical stretches) in the Neutral State and the Anionic State, supplemented by DFT calculations. Our focus is on the observed redshift, which we attribute to the weakening of the bond after receiving an electron from the donor. This is consistent with our experimental findings.

When analyzing the DFT calculations for the CN vibrational band in three different states, we note a consistent trend similar to what we observe for the C=O bond (as displayed in Figure 1Sc in the corrected version). To comprehensively address this question, we have made adjustments to Figure 4d (see Figure below). Additionally, we have included the value of the CN vibrational frequency in the ground state, enabling readers to assess the data independently.

In response to the second aspect of the reviewer's inquiry, we have provided a partial answer within question (c) of our response letter. In this section, we introduced an additional experiment involving transient mid-IR spectroscopy for IEICO-4F under 730-nm excitation. We highlighted that the configuration of the CN band observed under both 450 nm and 730 nm excitations exhibits similarity. This similarity signifies that the material's absorption at 450 nm is indeed adequate for excitation. In addition, we have expanded the discussion in the 'Introduction' section on page 2 of the revised manuscript and included two references kindly suggested the reviewer [1] and [2].

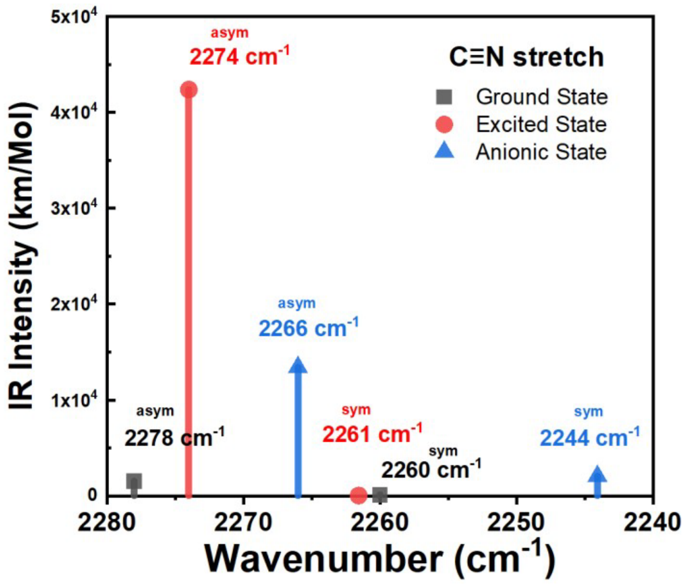

My careful review of the authors' responses and the mid-IR spectroscopy that is presented has raised several additional questions/concerns that the reviewers should also address:

1. The statement that the authors make on p. 2 is problematic:

"The calculations reveal that electron injection weakens the C≡N bond character in the acceptor material". The authors do not indicate clearly which band in Figure 3e and 3f is the symmetric and which is the asymmetric stretch. Regardless, one of these bands is higher in frequency than either band in the ground electronic state of the neutral (Figure 3d). How can the C≡N be weaker if one of its modes is blue shifted to a higher frequency?
The authors must justify this carefully, and they must show more of the assignments from their DFT calculations, also for anions, and show where the symmetric and asymmetric stretches are predicted to occur.

All mid-IR fs spectra in Figure 3(d-f) show a sharp drop near 2300 cm-1. Have the authors considered whether they are accurately able to measure these signals, which fall near the edge of their detection window? They should test by re-centering their mid-IR probe pulse at 2300 cm-1 and comparing transient spectra measured there with the ones shown in the paper.

Response: We thank the reviewer for the remark. In the revised version of Figure 4d, we have thoughtfully assigned designations to each CN vibrational stretch, denoted as "sym" (symmetrical) and "asym" (asymmetrical). The reviewer has also raised a parallel concern as previously articulated in question/section g, centered on the blue shift in CN vibrational bands. In our manuscript, we conduct a comparison between the Neutral and Anionic States, subsequently elucidating the peak positions. It is within this context that we address the redshift phenomenon (or weakening of CN band), characterized by the transition from Neutral States to Anionic States.

We acknowledge the potential for confusion that may arise from this approach and have taken proactive measures to rectify this concern. Specifically, we have undertaken revisions in the manuscript's updated

version to modify and expand the discussion in section Mid-IR fs Spectroscopy for $FAPbBr_3$ and $FAPbBr_3$/IEICO-4F on page 6.

In response to the second aspect of the reviewer's inquiry, the choice of the spectral range from 2075 cm^{-1} to 2300 cm^{-1} was carefully selected, aimed at facilitating a direct comparison between Figures 3d, e, and f. This approach was intended to illustrate the distinctions among the Neutral state of IEICO-4F film, Anionic states at 450 nm, and other Anionic states excited at 350 nm in $FAPbBr_3$/IEICO-4F film. To fully address the reviser's concern, we repeated the mid-IR measurements for IEICO-4F film in the range 2180 – 2350 cm^{-1}, where the Excited CN band has been centered in the given region (see Figure below). We are pleased to confirm that the observed CN band in this expanded range remains consistent with the presentation in the manuscript. We have expanded the discussion on page 6 and included Figure S6 in the Supplementary Information to provide additional insights and data.

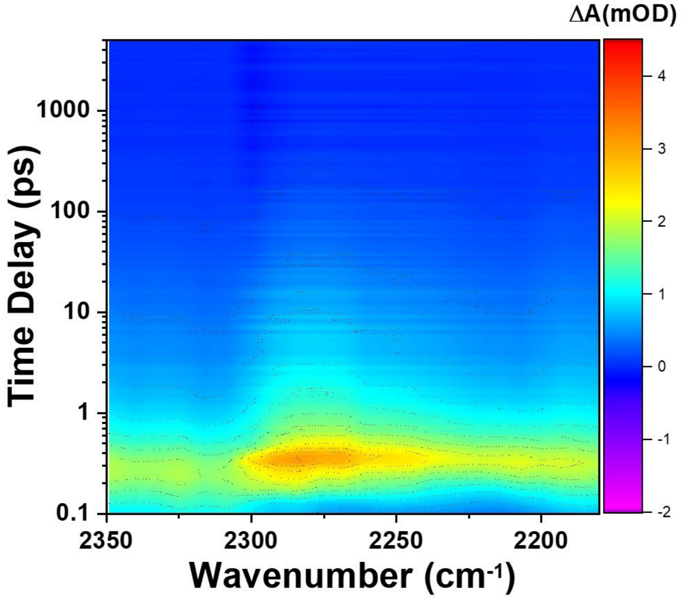

2. The DFT calculations show a tremendous difference in intensity of the symmetric vs. asymmetric stretching modes of the nitrile group (Fig. 4d). This is not matched by the more similar amplitudes in Fig. 3e,f. Also, the splitting is ~100 cm-1 in the experiments, but only ~10-20 cm-1 in the calculations. In agreement with the calculations, there is a small ~10 cm-1 splitting for the ground state of IEICO-4F and a very weak shoulder (Fig. 1d). The authors' calculations in Figure 4d reproduce neither the experimentally observed splitting nor the amplitudes seen in the experimental spectra. If so little about the calculated spectrum matches the experiment, then how can the authors' assignment be justified? The authors must address this.

Response: We thank the reviewer for the valuable comment. In the literature (JCTC, 2010, 6, 828), it was noted that B3LYP-calculated vibrational frequencies exhibited significant deviations from experimental values, particularly in open-shell systems. Consequently, CCSD(T) methods with triple-ζ basis sets were suggested as a more accurate alternative for vibrational frequency calculations. DFT calculated frequencies differ from experimental values due to the lack of anharmonicity in the model. The anionic form of IECO-4F is indeed an open-shell system, where B3LYP calculations can deviate from experimental

values by as much as 100 cm^{-1}, as shown in the paper. However, it's worth noting that the application of CCSD(T) methods to large molecules like

IEICO-4F is not feasible. On the other hand, it was shown that the calculated infrared (IR) intensities using density functional theory have been in good agreement with the experimental data and high-level ab initio correlated methods (J. Chem. Phys. 109, 10587–10593 (1998)). We looked at the calculated vibrational frequencies at 2280 and 2304 cm^{-1} and found that they correspond to symmetric and asymmetric C-N stretch, and the intensities are in line with experimental observation. Now, we have modified the sentence "fully supported by DFT" in the abstract with "align with the density functional theory (DFT) calculations." and "Our B3LYP-calculated vibrational frequencies are deviated from experimental values due to the lack of anharmonicity in the model as re-ported in the literature". In order to comprehensively address this question, we have expanded the discussion in the 'DFT calculations' section (page 7) and included the referenced material to provide additional context and support.

3. Can the authors more clearly explain how ns PL lifetimes are observed even though charge injection takes place on a sub-ps timescale? A clear explanation is warranted for the general readership of ACS Central Science.

Response: We appreciate the reviewer's comment. The TR-PL and mid-IR-TA measurements complement each other, providing distinct types of information. Charge transfer exhibits temporal diffusion, characterized by both short and long-lived components. In our experiments, mid-IR measurements primarily capture sub-ps charge injection dynamics. In contrast, due to the temporal resolution limitation of the TCSPC technique, it only detects the long-lived component which could be due to the long distance charge transfer and recombination, manifesting as an effect on PL lifetime within the ns timescale. To further elucidate this issue, we have expanded the discussion in the 'Results and Discussion' section of the main text (refer to page 2) and added the appropriate reference.

Minor issues:

M1. In Figure 1d the authors illustrate a vertical scale multiplier for the middle portion of the FTIR spectra, but the number in front of the 'x' character is missing for the black curve. If the nitrile stretch is unchanged, then is the scaling 3.5 x as in the red spectrum?

Response: We thank the reviewer for this remark. The answer is "Yes", the scaling is 3.5 x in the red spectrum. The missing number is '1' in front of 'x'. As requested, we added this number in Figure 1d for the black curve.

M2. Figure 4c. The authors do not state what quantity is shown by the color bars and what the units are.

Response: We appreciate the reviewer for highlighting this particular aspect. We confirm that the units displayed by the color bars are atomic units (a.u.). In accordance with your suggestion, we have included the units explicitly in Figure 4c. We also expanded the discussion about the given units on page 7 in the DFT calculation section. M3. Reviewer 2 is correct that the authors should state their IRF in the paper. The authors communicated it to the reviewers, but it should be in the

Response: We thank the reviewer for pointing this out. We agree with the reviewer on the importance of showing the IRF in the paper. We have expanded the discussion about the IRF of the TA measurements on page 8 in the Experimental Methods section.

oc-2023-00562u.R3

Name: Peer Review Information for "Real-Time Tracking of Hot Carrier Injection at the Interface of FAPbBr3 Perovskite Using Femtosecond Mid-IR Spectroscopy"

Third Round of Reviewer Comments

Reviewer: 1

Comments to the Author

The authors have responded to nearly all of my comments suitably (but see below for an important exception), and the addition of the new measurements with excitation at 730 nm is appreciated. The manuscript is now acceptable for publication in my view, but I urge the authors to respond to a comment from the last round of review that they ignored. The authors, to my knowledge, have not indicated what scaling factor was used with their computed IR frequencies. They should do so, or their work cannot be considered to be reproducible / adequately described. It is commonplace to use a scaling factor when publishing computed vibrational frequencies. The authors must either state the scaling factor they used or explicitly state that unscaled frequencies are presented.

Author's Response to Peer Review Comments:

KING ABDULLAH UNIVERSITY OF SCIENCE AND TECHNOLOGY

Omar F. Mohammed, PhD, FRSC, FIMMM
Associate Editor for ACS Applied Materials & Interfaces
Professor of Chemistry and Material Sciences and Engineering
Advanced Membranes & Porous Materials Center & KAUST Catalysis Center
Division of Physical Sciences and Engineering
Room 3277, Level 3, Building 5
KAUST, Thuwal 23955-6900, KSA
Phone: +966 2 808 4491

October 10th, 2023

Thank you for your email on October 5th, 2023, regarding our manuscript oc-2023-00562u.R2. We are grateful again to the referee for recommending publication in *ACS Central Science* after minor revision. In addition, we thank you so much for giving your attention to this work. The reviewer raised minor-clarification comment to improve the presentation of our manuscript, which we have fully addressed in the revised version of the manuscript. You will see in the next paragraphs our response to the reviewers' comment. Furthermore, we have made the necessary formatting adjustments in accordance with the editor's guidelines, and these changes can be found in the revised version of the manuscript.

Finally, I hope that you will find our revised manuscript both compelling and publishable in *ACS Central Science*. I look forward to hearing from you soon.

Best regards,

Omar F. Mohammed, PhD, FRSC, IOP, FIMMM

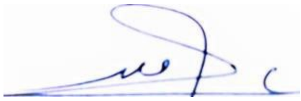

2019 & 2020 & 2021 & 2022 highly cited researcher (Web of Science) Associate Editor for ACS Applied Materials & Interfaces

KING ABDULLAH UNIVERSITY OF SCIENCE AND TECHNOLOGY

Division of Physical Sciences and Engineering, Thuwal 23955-6900, KSA

Reviewer: 1

The authors have responded to nearly all of my comments suitably (but see below for an important exception), and the addition of the new measurements with excitation at 730 nm is appreciated. The manuscript is now acceptable for publication in my view, but I urge the authors to respond to a comment from the last round of review that they ignored. The authors, to my knowledge, have not indicated what scaling factor was used with their computed IR frequencies. They should do so, or their work cannot be considered to be reproducible / adequately described. It is commonplace to use a scaling factor when publishing computed vibrational frequencies. The authors must either state the scaling factor they used or explicitly state that unscaled frequencies are presented.

Response: We would like to express our gratitude to the reviewer for acknowledging the effort we have put into addressing the concerns raised earlier, and we greatly appreciate the recommendation of our manuscript for publication in ACS Central Science after minor revision. We also sincerely appreciate the reviewer's additional concern regarding the scaling factor of IR frequencies, as this is essential to enhance the clarity of our manuscript. We would like to clarify that in our IR frequency calculations, we utilized unscaled harmonic vibrational frequencies. We have clarified this issue in the main text in the Experimental Methods of the revised version of the manuscript (see page 8).

Formatting Needs:

AU EMAIL: Please include the email address of the corresponding author on the first page of the manuscript, and the Supporting Information if submitted, with an asterisk next to their name in the author list. Please be sure to label "email."

Response: As requested, we have incorporated the email address of the corresponding author on the first pages of both the Manuscript and the Supporting Information.

SI PARAGRAPH: If the manuscript is accompanied by any supporting information for publication, a brief description of the supplementary material is required in the manuscript. The appropriate format is: Supporting Information. Brief statement in non-sentence format listing the contents of the material supplied as Supporting Information.

Response: We have added the '**Ⓢ Supporting Information'** to the MS manuscript as requested.

SYNOPSIS MISSING: The synopsis should be no more than 200 characters (including spaces) and should reasonably correlate with the TOC graphic. The synopsis is intended to explain the importance of the article to a broader readership across the sciences. Please place your synopsis in the manuscript file after the TOC graphic, and label it as "Synopsis."

Response: Please find the following synopsis after the TOC graphic. 'This study explores and deciphers the ultrafast hot electron dynamics at the interface between perovskite and electron transporting layer using femtosecond visible and Mid-IR spectroscopy. SI PG#S: The supporting information pages must be numbered consecutively, starting with page S1.

Response: We have numbered the pages in Supporting Information starting with 'S1' and requested.
